# Social dilemma for 30 years: Progress, framework, and future based on CiteSpace analysis

**DOI:** 10.1097/MD.0000000000041138

**Published:** 2024-12-27

**Authors:** Juan Gao, Yuqing Geng, Xinying Jiang, Jianyi Li, Yan Yan

**Affiliations:** a School of Business, Shanghai Dianji University, Shanghai, China; b Nursing Department, Guizhou Nursing Vocational College, Guizhou, China.

**Keywords:** bibliometrics, CiteSpace, dynamics, evolution, social dilemma

## Abstract

Social dilemmas have been a popular research topic in the past 30 years, yet there is still a lack of interdisciplinary reviews. This study represents the first attempt to conduct a bibliometric analysis of social dilemma research over the past 30 years, aiming to identify the research status, research hotspots, and future trends in this domain. We conduct an interdisciplinary analysis of 3630 articles from 1993 to 2023 using CiteSpace software. We find that: (1) this research domain exhibits a fluctuating upward trend and possesses evident interdisciplinary characteristics. (2) Collaboration among authors, institutional and regional, is much more prevalent, especially in the evolutionary dynamics of human behavior, cooperation, and reinforcement learning. (3) The current hot trend in this field of research is to investigate the influencing factors and solutions for social dilemmas. Researchers have shown great interest in value orientation, social norms, fairness, punishment, and rewards in promoting cooperation. (4) In the future, this field will cover different disciplines, develop theoretical frameworks grounded in bounded rationality, explore the boundary conditions of effective strategies, and integrate emerging technologies. This study serves as a valuable reference for scholars seeking to navigate social dilemma research while also providing insights for managers aiming to devise practical solutions to social dilemmas.

## 1. Introduction

“Social dilemmas” or “collective action problems” refer to such conflict situations where individuals may prefer noncooperation to attain superior outcomes (often short-term). However, if all pursue this noncooperation course of action, the common interests are compromised (often in the longer term).^[1–3]^ Examples of such dilemmas include antibiotic-resistant diseases (superbugs), climate change, global financial crises, transnational terrorism, nuclear weapons proliferation, and preserving unique habitats.^[4–6]^ For instance, the indiscriminate use of antibiotics in patients who do not require them for faster recovery can lead to antibiotic resistance in bacteria, leaving patients who actually need treatment without access to effective drugs. Antimicrobial resistance is forecast to cost >10 million lives per annum by 2050, exceeding deaths from all forms of cancer combined. These dilemmas threaten societal well-being, underscoring the growing imperative for countries to address air environment, social conflicts, and employment problems.^[7–10]^ Relevant scholars have increasingly focused on the social dilemmas over the past 2 decades, offering numerous coping strategies for governments and social institutions to address this concern effectively.

Both social dilemmas and collective action problems refer to situations where individual rationality leads to collective irrationality.^[2]^ The collective action problem mainly deals with how large groups struggle to achieve collective goals due to individuals’ free-riding behavior.^[1]^ Classic examples include the tragedy of the commons and the prisoner’s dilemma. On the other hand, the concept of “social dilemmas” introduced by Dawes RM in 1980, which describe the conflicts arising from individuals’ pursuit of short-term interests while disregarding long-term collective interests at the societal level, highlighting the impact of individual decisions on society as a whole.^[11,12]^ From the perspective of the research field, social dilemmas encompass all situations where personal interests conflict with collective interests. These include collective action problems and other issues of conflict and cooperation in social interactions, such as trust and cooperation in international relations. Its scope of research and application fields is relatively broader. Therefore, we uniformly use the concept of social dilemma in this paper.

This study aims to conduct a bibliometric analysis of the authors, countries, and institutions involved in researching this topic. Furthermore, cluster analysis will be conducted on references and keywords to identify research hotspots and future directions. Before conducting the bibliometric analysis, we conducted a preliminary literature review of this research field. The social dilemmas were comprehensively explored in 2 distinct sections. The first section focuses on categorizing social dilemmas, while the second section explores potential solutions for addressing them.

### 1.1. The categories of social dilemmas

In the past 3 decades, researchers have often used the game theory paradigm to describe dilemmas in real society. There are two-person and multiple-person dilemmas based on the number of participants.^[13]^

Two-person dilemmas include classic scenarios like the prisoner’s dilemma, the chicken dilemma, and the stag hunt dilemma. The prisoner’s dilemma is characterized by a single equilibrium point known as mutual betrayal and is frequently utilized to simulate various real-world scenarios, including environmental protection, the tragedy of the commons, resource wastage, and price wars within society.^[14,15]^ The chicken dilemma, also known as the hawk-dove or snowdrift game, features 2 equilibrium points where one player betrays while the other cooperates.^[16,17]^ This dilemma describes a scenario where competition for a shared resource and the contestants can opt for either conciliation or conflict. The stag hunt dilemma, sometimes called the assurance game, trust dilemma, or common interest game, presents 2 equilibrium points (full cooperation or complete betrayal).^[18]^ The 3 dilemmas simulate real-life decision-making scenarios, covering cooperation and betrayal, high-risk and low-risk choices, competition, and adaptation. These scenarios provide a theoretical framework for analyzing and resolving complex problems.^[19–22]^

In addition to two-person dilemmas, researchers explore multi-person dilemmas such as public goods and common resource dilemmas.^[23,24]^ Public goods will not be available if no one chooses to contribute, resulting in the denial of benefits for all. However, if only some people prefer to contribute, they will bear the total cost, while others can benefit without contributing.^[25,26]^ Common resource dilemmas emerge when individuals collectively decide how to manage shared resources, including natural resource management and waste. Due to the non-excludable nature of public resources, individuals may tend to exploit them excessively, resulting in their depletion and environmental degradation. The simulations of public goods and common resources dilemmas reflect real-life situations, highlighting the inherent conflict between individual and collective interests. This not only deepens our understanding of how human society operates but also helps us develop better cooperative frameworks to improve the efficient use of public goods, thereby promoting sustainable resource utilization and environmental protection. In addition, some studies on social dilemmas do not use the game theory paradigm but field studies, laboratory experiments, and cross-community studies.^[27]^

### 1.2. Solving social dilemmas

#### 1.2.1. The theory of social dilemma

Social dilemma research involves several disciplines and theories. The following are some of the main relevant theories:

(1) Game theory: Game theory studies the strategic choices of rational individuals by constructing games and posits that economic rationality drives human behavior. Game theory includes the prisoner’s dilemma, tragedy of the commons, and public goods game.^[18,28]^ Different selection strategies have been proposed and examined in the current study. For example, in game theory, people employ strategies such as tit-for-tat, forgiving tit-for-tat, win-stay, and lose-shift.
(2) Evolutionary theory: Evolutionary theory explains human cooperation in social dilemmas from the perspective of natural selection. This theory includes kin selection, reciprocal altruism, group selection, and network reciprocity to explain cooperative behavior in social dilemmas.^[29–32]^ These various models based on evolutionary theory can provide rational explanations for individuals’ behavior across a wide range of social dilemmas.
(3) Bounded rationality: This theory emphasizes the adaptability of people’s behavioral choices in social dilemmas. For example, Ostrom E proposed the theory of autonomous governance, which solves collective action problems, and defined 8 principles.^[33]^ Perc M focuses on studying the interactions among individuals, groups, and even societies within social dilemmas, using evolutionary game theory as its framework.^[34]^
(4) Social interdependence theory: This theory posits that interdependence situations depend on 3 factors: the structure of interdependence (such as the prisoner’s dilemma), the parties involved (such as Player A and Player B), and the dynamics of interaction (such as the use of a tit-for-tat strategy). In interdependent situations, people focus on their own outcomes and those of others involved. Furthermore, people’s relative preferences for their own and others’ outcomes are stable.^[35]^
(5) Social value orientation: The theory explains individual preferences in resource allocation, encompassing self-interest, altruism, cooperation, and competition.^[36,37]^ Different social value orientation types significantly influence decision-making in social dilemmas.

#### 1.2.2. Strategies for solving social dilemmas

(1) Punishment: By punishing noncooperative behavior, such as imposing fines, demotion, or social exclusion, selfish actions are deterred and reduced.^[38]^ Punishment involves retaliation or negative reciprocity, where an individual incurs a cost to impose a cost on someone else. In peer punishment, individual players independently punish defectors.^[39]^ In pool punishment, those willing to punish contribute to a common pool, from which resources are drawn to punish defectors when necessary.^[34]^
(2) Reward: Rewarding is prevalent in human societies as a form of positive reciprocity towards well-behaved and prosocial actions.^[40]^ Unlike punishment, rewarding involves incurring a cost to benefit someone else. Individuals personally reward other cooperators or contribute to a common pool used to reward cooperators. Similar to punishment, rewarding aims to influence recipients into actions that benefit the rewarders.
(3) Communication: Many studies have shown that communication helps increase cooperation in social dilemmas.^[1]^ When individuals have the opportunity to communicate with each other, the level of cooperation increases significantly.^[41]^ On one hand, the process of communication facilitates understanding others’ desires. When individuals know whether others will cooperate or betray in a social dilemma, they can adjust their coping strategies accordingly. On the other hand, communication allows individuals to express themselves and make a clear commitment to their intentions. This commitment can enhance cooperation levels in challenging social situations.
(4) Reputation: Having a good reputation is crucial for establishing an individual’s social identity and earning the trust of others. Previous research shows that groups with a reputation mechanism have higher cooperation levels than those without.^[42,43]^ Reputation is based on past behavior, and even in the absence of current information, we can determine whether an individual is more likely to cooperate or betray by referring to historical records. The essence of a reputation system lies in establishing a trust relationship through repeated interactions between both parties. In addition, even if the 2 parties do not have any direct connection, they can indirectly establish a relationship of trust through the reputation they have previously established with others.
(5) Reciprocity: Reciprocity has always been regarded as an important motivation in human behavior, including direct and indirect reciprocity, which can explain cooperation in many social dilemmas.^[44]^ In a social dilemma, helping someone well leads to an expectation of reciprocal treatment. When the other person promises or reciprocates, this is known as direct reciprocity. Additionally, indirect reciprocity refers to situations where an individual’s cooperation and assistance towards another person cannot be directly rewarded by that person but can only be indirectly rewarded through a third party or other means. Both direct and indirect reciprocity have contributed to promoting cooperation and achieving mutually beneficial outcomes.^[45]^
(6) Fairness: Fairness is a social norm that has evolved throughout human history and is an essential criterion for individuals when making choices in social dilemmas.^[46]^ Some studies have explored the influence of distributive fairness on cooperative behavior in public resource games, and the results show that individuals who perceive fairness exhibit more cooperative behavior than those who perceive unfairness under equal conditions of effort and contribution.^[47]^ Regarding organization, the fairness of distribution results is significantly and positively correlated with job satisfaction among organizational citizens,^[48]^ trust in authority, organizational commitment, and other intentions of organizational citizenship. These findings demonstrate that maintaining equity is crucial for effectively promoting cooperation.

To summarize, scholars have conducted extensive research on social dilemmas and their solutions over the past 30 years. As the number of research findings in social dilemmas grows, some researchers have authored comprehensive review articles to summarize these results. Some reviews adopt various disciplinary perspectives, encompassing psychology, statistical physics, and economics.^[18,28,34]^ Others have focused on summarizing outcomes from diverse social dilemmas research themes, such as game theory, social norms, the tragedy of the commons, and climate change.^[5,18,49–51]^ While these reviews from specific perspectives help researchers understand social dilemma issues, they often lack interdisciplinary and comprehensive research analyses in this area. This gap impedes future researchers from achieving academic and systematic integration in this research field.

In order to cover the research gaps, this paper uses CiteSpace bibliometric software to reorganize the literature on social dilemmas. A knowledge map was constructed to describe the knowledge foundation, cooperation, status, and evolution. Future research directions were provided accordingly. This information will help researchers broaden the research perspective and improve future research effectiveness. Additionally, by summarizing research on social dilemmas, we can identify factors influencing the resolution of social dilemma issues, seek strategies for addressing them, and provide evidence for organizations and governments to formulate relevant policies. The following 4 key questions are the focus of this paper.

Question 1. What are this research field’s publication status and trends in the last 30 years?
Question 2. What research topics have institutions, regions, and authors collaborated on in the past 30 years?
Question 3. How has the research on social dilemmas changed and evolved in the last 30 years?
Question 4. What are social dilemmas’ top topics and research frontiers in the past 30 years?

**Figure 1.**
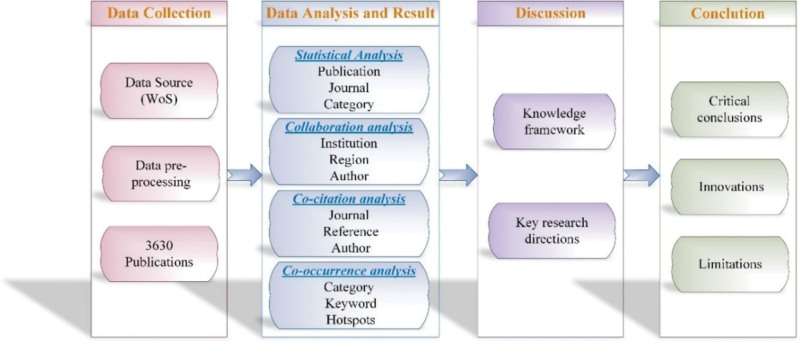
Research process.

The remaining parts of this article are organized as follows. Section 2 introduces the research methodology of this article, explaining the data sources and selection process. Section 3 conducts a data visualization analysis on 3630 documents and presents the result (the primary characters of the literature, the collaboration networks, the co-citation networks, and the co-occurrence networks). Based on the above findings, section 4 conducts a knowledge framework and points out key research directions. The last section is the conclusion. The research process is depicted in Figure 1.

## 2. Methods

### 2.1. Analysis method selection

*Bibliometrics* is a statistical method used to evaluate and monitor the progress of specific academic fields by sorting data. This method enables us to gain a clear and objective understanding of the development of research topics by analyzing patterns in academic literature, such as document count, authorship, and keywords.^[52,53]^ Researchers can quantitatively evaluate the influence and quality of research topics by analyzing evaluation indicators such as references’ citation frequency, journal impact factor, etc, to uncover the trends in the development of research topics.^[54]^ Bibliometric software enables researchers to generate knowledge maps visually illustrating the citation relationships among literature nodes on various topics.^[55]^ This facilitates researchers’ intuitive understanding of the correlation and academic inheritance between literature and the differentiation of distinct research directions.

Several software and methodologies are available for quantitative bibliometric analysis of extensive publications. Examples include HistCite, VOSviewer, Biblioshiny, and CiteSpace.^[56–58]^ In this study, we not only need the software to have basic statistical functions but also require it to possess the capability of analyzing regional, institutional, and author cooperation; literature and author co-citation; keyword co-occurrence; as well as conducting timeline analysis. The CiteSpace software (version 6.2R3) was chosen for our study after comparing the software functions, operability, and visualization effects among different statistical software. HitsCite relies solely on word frequency analysis and does not provide insights into author, reference, and keyword relationships. It limits its utility for co-occurrence, co-citation analysis, and identifying collaboration networks.^[59]^ The operation of VOSviewer is simple, but the generated graph style is relatively basic and cannot display timeline maps.^[60,61]^ Biblioshiny can import data from various sources, such as Scopus and Web of Science, and provides different types of bibliometric analysis. However, it produces fewer visualizations compared to CiteSpace.^[62]^ CiteSpace’s advantage lies in its comprehensive functionality, making it a suitable choice for our analysis. Scholars have used this software to visually analyze the characters of literature, collaboration networks, co-citation networks, and co-occurrence networks related to sustainable tourism and clean energy production. This analysis provides a knowledge framework for subsequent researchers and policymakers.^[63,64]^ The details of CiteSpace software applied in this study are as follows:

(1) Cooperation analysis: We utilized CiteSpace to explore collaboration among various institutes, regions, and authors in social dilemma research. By generating visual cooperation networks, we gained insight into the specific research areas these groups collaborate on.
(2) Co-citation analysis: CiteSpace assisted in constructing a knowledge base by pinpointing the most influential journals, references, and authors in social dilemma research through co-citation analysis. This analysis enabled us to understand the impact of journals, references, and authors on recent studies.
(3) Timeline view: Using CiteSpace’s timeline feature, we could observe the publication dates and peak interest periods of articles and keywords in the research field. This visualization aided in tracking the evolution of research topics over time and identifying popular study areas.

### 2.2. Data sources

The Web of Science Core Collection database was used in this study to find articles on social dilemmas. The selection of this database was based on its reputation as a comprehensive academic resource with detailed information on authors, institutions, countries, journals, and cited references. Therefore, the literature data for this research were obtained from the Web of Science Core Collection, which indexes the Science Citation Index Expanded, Social Sciences Citation Index, and Arts & Humanities Citation Index. These 3 collections are widely recognized internationally and are considered the primary repositories for the high-quality scholarly papers in this field. This enables us to investigate the most representative papers and authoritative authors in the research domain of social dilemmas. Additionally, many bibliometric studies only rely on the Web of Science Core Collection as their primary source of literature, further validating this database’s accuracy. Furthermore, the current bibliometrics software is limited to analyzing papers from a single database, which presents challenges when conducting analyses across multiple databases. Therefore, we excluded other databases such as Scopus, Engineering Village, and PubMed.^[65]^

### 2.3. Selection process

As mentioned above, we have chosen the Web of Science Core Collection as the literature source to consider the quality and reliability of the literature.^[55,66]^ To determine this study’s “topic” keywords, we reviewed highly impactful literature in this research field and searched the Web of Science for synonymous academic keywords. After comparing each synonym’s conceptual scope and application field, we have found that both “social dilemma” and “collective action problem” revolve around the conflict between individual and collective interests, with overlapping application areas in recent years. Therefore, we have chosen them as the topic keywords for this study. We specifically used the following search strategies: topic = [social dilemma*] or [collective action problem*]; the search was limited by document type (article and review) and language (English). Note that the data collection date was June 3,2023. Additionally, we did not restrict the search to specific academic disciplines to ensure a comprehensive literature search on social dilemmas.

Two authors were engaged to conduct a comprehensive evaluation of the titles, keywords, and abstracts of these articles to ensure their content is related to social dilemmas.^[66]^ The primary exclusion criterion for topic keywords was “this paper does not pertain to any aspect of social dilemma research.” After screening the titles, abstracts, and details of the literature to ensure that it was relevant to social dilemmas or collective action problems, 2 authors deleted 3 pieces of literature of low relevance. Finally, 3630 articles were selected for the database in the 1993 to 2023 timespan. Subsequently, we employed deduplication in CiteSpace, and no instances of document duplication were detected. So, these records were used in this research as a sample.

## 3. Results

### 3.1. Publication statistical analysis

This sector aims to answer Question 1 by publication statistics to show this field’s primary publication status and trend, helping scholars know whether this field is popular and emerging. The publication of statistical analysis allows scholars to understand the primary categories of social dilemma research and what journals are more welcomed by scholars in previous studies.^[52]^

#### 3.1.1. Number of publications by years

**Figure 2.**
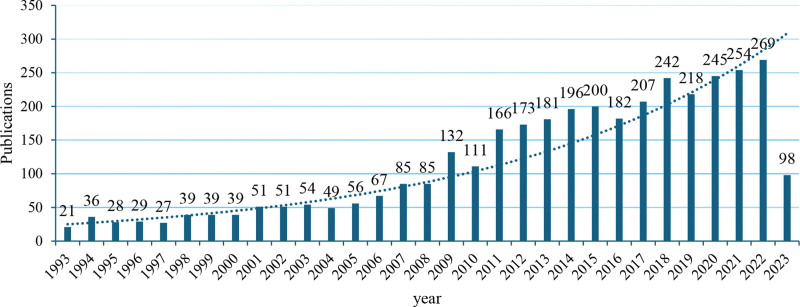
Number of publications by years.

Figure 2 illustrates the exponential growth in the number of articles on social dilemmas over the past 3 decades. The figure shows that annual publications exceeded 100 per year after 2009 and over 200 per year after 2015. In 2022, there were 269 publications, over 11 times the number in 1993 (count 21). Additionally, there are notable spikes in publications, particularly in 2009. This is because the researchers’ attention to social dilemma research has been heightened due to the impact of the 2008 financial crisis. The number of social dilemma articles surged to 2874 from 2009 to 2023, nearly quadrupling the 750 publications from 1993 to 2008. Considering ongoing challenges such as the depletion of natural resources, climate change, and political divisions, we anticipate a continued growth in publications in this research domain. It should be noted that the number of publications in 2023 was only 98 because the search was conducted on June 3, 2023, and only included part of the year’s publications. This means that our collection of articles published in 2023 is smaller than that of 2022, so it remains to be seen whether the number of articles in this research domain will continue to increase. In future studies, we will keep track of this year’s data further to analyze research trends in social dilemmas.

#### 3.1.2. Number of publications by journals

In general, we can assess the quality of a journal based on 2 factors: impact factor and journal rankings. The impact factor is essential for measuring a journal’s academic level and influence. Articles with higher impact factors tend to receive more frequent citations, indicating increased authority and significance within the field. Journal rankings can reflect the status and influence of a journal in the research domain because they are divided into 4 equal parts based on the previous year’s articles’ impact factor values, arranged in descending order from high to low, known as Quartile 1, Quartile 2, Quartile 3, and Quartile 4. Therefore, the journal rankings not only consider the impact factor but also consider the journal’s ranking in different subject areas, which reflects its relative position within the field and objectively indicates its level.

Over the past 30 years, 990 journals have published articles on social dilemmas. Table 1 illustrates the top 14 journals by number of publications. Journals with more than 90 articles include the multidisciplinary journals Scientific Reports (impact factor = 5.516, Quartile 2) and PLoS One (impact factor = 4.069, Quartile 2). There are other productive journals, including Proceedings of the National Academy of Sciences of the United States of America (impact factor = 13.451, Quartile 1), Chaos Solitons & Fractals (impact factor = 7.02, Quartile 1), Journal of Personality and Social Psychology (impact factor = 10.504, Quartile 1), and Organizational Behavior and Human Decision Processes (impact factor = 6.737, Quartile 1). Additionally, among the top 14 journals by article counts, 8 have an impact factor of 5 or below.

**Table 1:** Number of publications by journals.

Ranking	Journal	5-year impact factor	Counts	Journal ranking	Percentage (%)
1	Scientific Reports	5.516	94	Quartile 2	2.590
2	PLoS One	4.069	90	Quartile 2	2.479
3	Physica A: Statistical Mechanics and its Applications	3.135	77	Quartile 2	2.121
4	Chaos Solitons & Fractals	7.02	75	Quartile 1	2.066
5	Journal of Theoretical Biology	2.405	73	Quartile 3	2.011
6	Applied Mathematics and Computation	3.767	62	Quartile 1	1.708
7	Journal of Economic Behavior & Organization	2.495	61	Quartile 2	1.680
8	Organizational Behavior and Human Decision Processes	6.737	54	Quartile 1	1.488
9	Physical Review E	2.537	54	Quartile 1	1.488
10	Journal of Economic Psychology	3.585	48	Quartile 2	1.322
11	Rationality and Society	1.092	48	Quartile 4	1.322
12	Proceedings of the National Academy of Sciences of the United States of America	13.451	47	Quartile 1	1.295
13	Journal of Personality and Social Psychology	10.504	44	Quartile 1	1.212
14	Journal of Experimental Social Psychology	5.701	42	Quartile 2	1.157

From the above data, we can derive 2 insights. On the one hand, these data indicate that journals publishing articles on social dilemmas include top journals with higher impact factors and ordinary journals with lower impact factors. On the other hand, despite having lower impact factors, some journals rank high within their relevant disciplinary fields. The Applied Mathematics and Computation (impact factor = 3.767, Quartile 1) is an example. So, evaluating the quality of journals in this field should not solely rely on impact factors but also consider journal rankings. These findings provide valuable insights for researchers in this field when selecting journals.

#### 3.1.3. Number of publications by categories

Table 2 illustrates the top 10 categories by number of publications. We can find that 562 articles come from economics, 398 from social psychology, 318 from multidisciplinary science, 290 from political science, and 257 from multidisciplinary physics (15.482%, 10.964%, 8.760%, 7.989%, and 7.080%, respectively). In addition, environmental studies, management, sociology, and mathematical physics are also essential categories in the social dilemma research domain. These pieces of information illustrate this research domain’s interdisciplinary and comprehensive characteristics.

**Table 2:** Number of publications by categories.

Ranking	Category	Counts	Percentage (%)
1	Economics	562	15.482
2	Psychology Social	398	10.964
3	Multidisciplinary Sciences	318	8.760
4	Political Science	290	7.989
5	Physics Multidisciplinary	257	7.080
6	Psychology Multidisciplinary	252	6.942
7	Environmental Studies	243	6.694
8	Management	203	5.592
9	Mathematical physics	170	4.683
10	Sociology	161	4.435

Each discipline offers unique insights and methods, helping us better understand and solve social dilemmas. Economists concentrate on solving social dilemmas at both the government and societal levels. They investigate incentives, penalties, fairness, equity, and information transparency and how these impact individuals’ behavior in social dilemmas. Economists also craft policies and strategies to tackle and resolve these issues effectively. Psychologists focus more on explaining the psychological mechanisms and behavioral choices of individuals facing social dilemmas from cognition, behavioral motivation, and social norms. For example, they study how emotions (such as empathy, anger, guilt), social cognition (social identity, social recognition, group belonging), and motivation (intrinsic and extrinsic) influence people’s behavioral choices in social dilemmas. Sociophysicists predominantly employ physical methods and models to simulate the behaviors and interactions of individuals and groups across different social dilemmas. They investigate how interactions among individuals give rise to group behaviors, analyze the evolution of cooperative behavior, and examine the factors contributing to resolving social dilemmas.

### 3.2. Collaboration analysis

This sector aims to address Question 2 through collaboration analysis to explore collaboration networks, illustrate collaborative relationships, and identify key research topics among institutions, regions, and authors. Collaboration analysis can help scholars seek potential collaborators, understand overall characteristics, and gain valuable insights into previous studies.^[67]^

#### 3.2.1. Institution collaboration

Table 3 exhibits the top 18 collaborating institutions among the 535 institutions researching social dilemmas. We can find that 12 institutions are in Europe, 2 in the United States, 2 in China, and 1 in Japan. These data show that European institutions pay more attention to social dilemmas. This is because Europe has more countries facing an aging population, energy crises, and local conflicts; exploring the formation and solution of these social dilemmas is a priority for Europe’s institutions.^[44]^ Additionally, the top 3 research institutions in terms of publication numbers are the Max Planck Society (counts 124, centrality 0.11), the University of California System (counts 115, Centrality 0.17), and RLUL-Research Libraries (counts 93, Centrality 0.12). These 3 research institutions publish many articles on social dilemmas and have high centrality values above 0.1. It is indicated that these institutions play a crucial role in this research domain because the node’s centrality is proportional to the node’s impact. These findings help scholars to know which institutions are more likely to cooperate in this research domain.

**Table 3:** Institution collaboration.

Ranking	Institution	Country	Counts	Centrality	Year
1	Max Planck Society	Germany	124	0.10	2002
2	University of California System	America	115	0.17	1996
3	RLUL-Research Libraries	UK	93	0.12	1997
4	University of Maribor	Slovenia	87	0.09	2009
5	Harvard University	America	84	0.12	1999
6	University of London	UK	83	0.13	1997
7	Tilburg University	Netherlands	77	0.03	2005
8	Eotvos Lorand Research Network	Hungary	73	0.02	1999
9	Hungarian Research Centre for Natural Sciences	Hungary	68	0.02	1999
10	Vrije Universiteit Amsterdam	Netherlands	59	0.06	1995
11	The Hungarian Academy of Sciences	Hungary	57	0.01	2012
12	North western Polytechnical University	China	53	0.01	2017
13	Leiden Universiteit	Netherlands	53	0.01	1995
14	Leiden Universiteit-Excl LUMC	Netherlands	53	0.01	1995
15	Kyushu University	Japan	51	0.03	2009
16	N8 Research Partnership	UK	49	0.07	1999
17	Peking University	China	46	0.04	2009
18	University of Oxford	UK	46	0.03	2009

**Figure 3.**
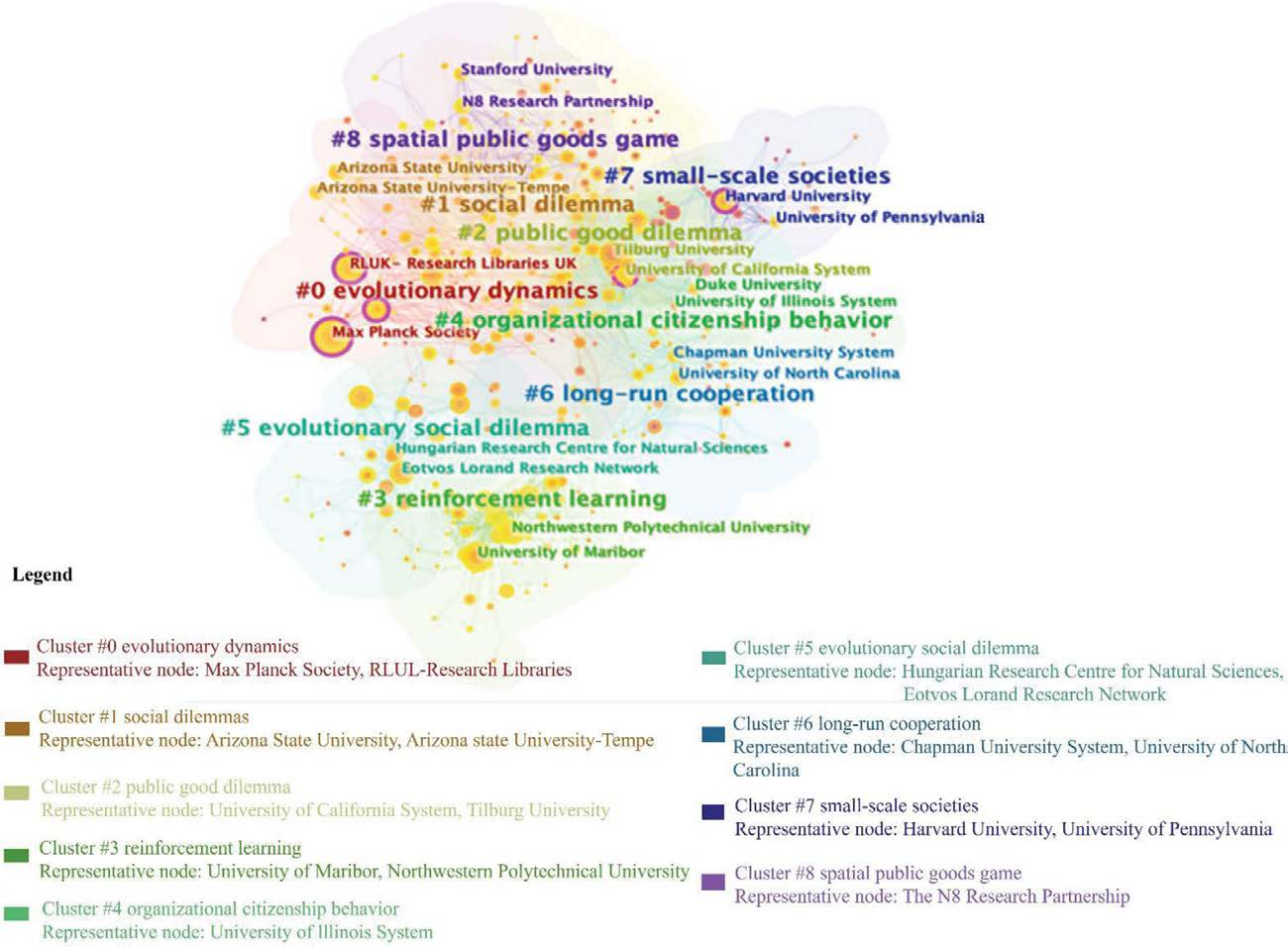
Institution collaboration clusters (nodes = 536, links = 2038).

Figure 3 depicts the cluster view of the institutional collaboration network with 536 nodes and 2038 links. Node size corresponds to the institution’s article counts, while links represent collaborative relationships among institutions. Notably, the purple rings around specific nodes indicate their crucial role in connecting different parts of the networks (high centrality), with the thickness of the rings reflecting the strength of this centrality. The institution’s collaborations are further categorized into 9 clusters. Specifically, clusters involving institutions with 50 articles or more are outlined below:

1. Researchers from the Max Planck Society and RLUL-Research Libraries focus on evolutionary dynamics (#0).
2. Researchers from the Arizona State University and Arizona State University-Tempe revolve around social dilemmas (#1).
3. Researchers from the University of California System and Tilburg University concentrate on the public good dilemma (#2).
4. Researchers from the University of Maribor explore efforts to reinforcement learning (#3).
5. Researchers from the Hungarian Research Centre for Natural Sciences and Eotvos Lorand Research Network emphasize the evolutionary social dilemma (#5).
6. Harvard University researchers specialize in small-scale societies (#7).
7. The N8 Research Partnership researchers focus on the spatial public goods game (#8).

Additionally, institutional collaboration extends not only within clusters but also across clusters. For instance, the Max Planck Society has collaborated with the University of California System on research involving evolutionary strategies for navigating social dilemmas.^[45,68,69]^ It has also partnered with Vrije Universiteit Amsterdam to address climate change issues.^[70,71]^ The Complex Systems Center of Maribor has collaborated with the Hungarian Academy of Sciences to study the impact of factors like intelligence, conformity, and aging on the evolution of social dilemmas for several decades (2008–2019).^[72–78]^

In conclusion, the evolutionary dynamics of people’s behavior, cooperation, and reinforcement learning are critical topics in institutional collaboration within social dilemmas. The public goods dilemma and spatial public goods game are primary paradigms in this research domain. As a result, scholars seeking cooperation opportunities should consider that different institutions may possess diverse specialized research interests when collaborating.

#### 3.2.2. Region collaboration

In Table 4, 13 collaborating regions are listed, each having conducted social dilemma research and published over 100 articles. These regions include 2 North American countries (the United States and Canada), 2 Asian countries (China and Japan), 1 Oceanian country (Australia), and 8 European countries. Notably, the United States has the highest number of articles (1307 counts), initiates research earliest (1990), and holds the highest centrality (0.46). This information signifies the United States’ leading position in social dilemma research. Furthermore, China is the only developing region in the top 13 list. Despite its developing status, China has maintained a consistently high economic growth rate since 1990.^[79]^ Previous studies have also shown that the higher the level of economic development, the greater the financial support for academic research provided by the state, and consequently, scholars produce more academic output.^[80,81]^ Additionally, the centrality of Japan (177 articles) and Australia (122 articles) are notably low at 0.02. Lower centrality suggests these regions could strengthen collaboration with others to increase their influence in this research domain.

**Table 4:** Region collaboration.

Ranking	Region	Counts	Centrality	Year
1	USA	1307	0.46	1993
2	China	504	0.08	1998
3	Germany	451	0.27	1998
4	England	408	0.20	1995
5	Netherlands	366	0.25	1995
6	Japan	177	0.02	1995
7	Switzerland	141	0.06	1997
8	Canada	138	0.06	1996
9	Italy	129	0.07	1999
10	Australia	127	0.04	1997
11	Austria	125	0.02	1998
12	Sweden	124	0.06	1998
13	Spain	116	0.08	2001

**Figure 4.**
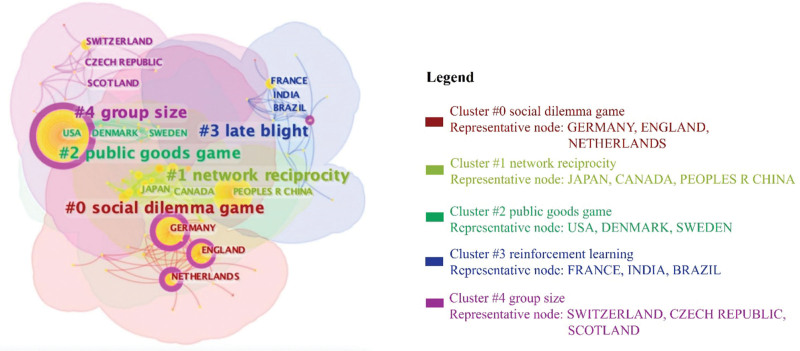
Region collaboration clusters (nodes = 97, links = 640).

Figure 4 illustrates the cluster of regional collaboration networks with 97 nodes and 640 links. The regional collaborations are categorized into 5 clusters. Specifically, researchers from Germany, England, and the Netherlands primarily focus on the social dilemma game (#0); researchers from China and Japan emphasize network reciprocity (#1); researchers from the USA, Denmark, and Sweden initiate research on the public goods game (#2); researchers from France, India, and Brazil appears more inclined towards studying late blight interrelated social dilemmas (#3); and researchers from Switzerland and the Czech Republic are concerned with the group size of social dilemmas (#4). The cluster of regional collaboration networks gives researchers a concise overview of the primary research focuses in each area. The cluster of regional collaboration networks gives researchers a concise overview of each region’s primary research focus and facilitates the discovery of potential collaboration partners.

#### 3.2.3. Author collaboration

Table 5 exhibits the top 13 authors who collaborated more than 19 times. The result shows that Perc M is the most collaborations contributing 87 articles, followed by De Cremer D (58), Szolnoki A (57), Wang Z (46), Tanimoto J (44), and Wang L (36). These scholars significantly contribute to social dilemma research through numerous collaborative articles. In terms of centrality, the top 4 authors are Nowak MA (0.06), Perc M (0.03), De Cremer D (0.03), and Van Dijk E (0.03), showing that these 4 authors play a critical role in connecting with other authors. For instance, De Cremer D and Van Dijk E collaborated over 15 times, exploring topics like self-benefiting in resource allocation, the impact of leader prototypicality on followers’ status, and the effects of endowment asymmetry on cooperation.^[46,81,82]^ Perc M and Szolnoki A, collaborating over 20 times since 2008, focus on group interaction patterns and environmental and individual factors affecting cooperative behavior in spatial social dilemmas.^[83–85]^ Based on the above analysis, most of the cooperative authors are statistical physicists and psychologists, and their articles are also the most. This result indicates that social dilemmas are an essential research topic in statistical physics and psychology.

**Table 5:** Author collaboration.

Ranking	Author	Counts	Centrality	Year
1	Perc, Matjaz	87	0.03	2008
2	De cremer, David	59	0.03	1999
3	Szolnoki, Attila	57	0.01	2008
4	Wang, Zhen	46	0.02	2011
5	Tanimoto, Jun	44	0.00	2010
6	Wang, Long	32	0.01	2007
7	Chen, Xiaojie	27	0.00	2007
8	Van Dijk, Eric	24	0.03	2006
10	Van Lange, Paul A M	19	0.01	2007
11	Traulsen, Arne	19	0.00	2010
12	Santos, Francisco C	19	0.00	2009
13	Nowak, Martin A	19	0.06	2008

**Figure 5.**
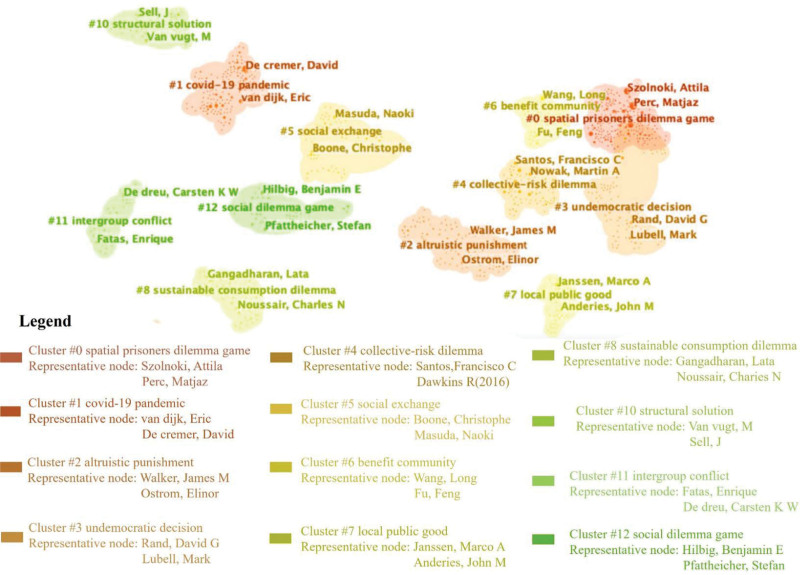
Author collaboration clusters (nodes = 4773, links = 7768).

Figure 5 shows the author’s collaboration visualization network, depicting 13 clusters based on the authors’ research topics. We classify these 12 clusters into 3 categories based on the varying levels of collaboration among authors. These 4 clusters mainly include social physicists and mathematicians, who mostly apply the method of building physical or mathematical models to study the evolution of cooperative behavior in social dilemmas and the influencing factors.

Firstly, there is apparent collaboration among authors within and between clusters with similar research topics or methodologies. This phenomenon is observed in cluster #0#, #3, #4, and #6. Authors in these clusters also tend to produce more articles.

(1) Spatial prisoner’s dilemma game (#0): It is the most collaborative cluster, led by Perc M and Szolnoki A, Wang Z, and Tanimoto J, focusing on using statistical physics methods to understand the evolutionary mechanisms and influencing factors of cooperation on social dilemma. In recent years, Wang Z team revealed how group size, communication, and behavior types drive prosocial behavior.^[41,86,87]^ Perc M research team explores the interaction between social mobility and network cooperation, discovering that various mobility ranges and probabilities have distinct impacts on cooperation.^[30,31]^ Furthermore, Perc M also partnered with Chen XJ (#6) and Wang L (#6) to explore the adaptive and bounded investment returns and generalized benefit functions’ impact on cooperation in spatial public goods games.^[76,88]^
(2) Undemocratic decision (#3): This cluster led by Rand DG and Lubell M. Rand DG research group explored cultural factors that influence individuals’ behavioral choices in social dilemmas, such as disseminating unjust information and selfish behavior.^[89,90]^ Rand DG also collaborated with Nowak MA and found that positive interactions and rewards are more effective in promoting cooperative behavior in public goods dilemmas.^[91]^
(3) Collective-risk dilemma (#4): Nowak MA and Santos FC lead this cluster. In collaboration with Pacheco JM, Souza MO, and Pinheiro FL, Santos FC studied the mechanisms of group fairness, social diversity, group members’ memories, and stress to promote cooperative behavior and how group fairness evolved.^[92–95]^ Nowak MA and Hibe C focused on memory strategies, zero-determinant strategies, and control in multi-person effects on social dilemmas and cooperation.^[96–98]^ Their recent collaboration investigates environmental information and strategies of direct reciprocity effects on social dilemmas and cooperation.^[99–101]^
(4) Benefit community (#6): Wang L is the most active author in this cluster. He cooperated with Su Q to explore the interactive diversity in the evolution of cooperation.^[102,103]^ Additionally, Wang JW research group found that the benefit community and willingness to cooperate promotes cooperation in social dilemmas.^[104,105]^

Secondly, there is apparent cooperation within the cluster, particularly in clusters #1, #5, and# 7. The researchers in these 3 clusters are primarily social scientists, such as psychologists, organizational behaviorists, and sociologists. They study social dilemmas from the perspective of organizational behavior.

(1) Corona virus disease 2019 (COVID-19) pandemic (#1): De Cremer D and Van Dijk are the most active authors in this cluster. They focus on organizational factors such as identification, leader behavior, endowment asymmetry, accountability, hierarchical position, and experienced power in social dilemmas from 2002 to 2018.^[105–108]^ Romano A. and Spadaro G. recently explored generational differences in behavioral responses to the COVID-19 pandemic. They found that older individuals perceive higher costs of virus infection but lower daily life costs during the flu season, while younger individuals have the opposite perception, potentially leading to intergenerational conflicts of interest.^[109,110]^
(2) Social exchange (#5): Yamagishi research team explored social exchange heuristics and found that a player in the prisoner’s dilemma is more likely to cooperate when perceiving the game as a social exchange.^[111,112]^
(3) Local public good (#7) Janssen Marco research team concentrates on public goods and resource dilemmas. They found that collective risks reduced people’s cooperation. Additionally, they investigated the combined effects of voting and enforcement on internalized motivation to cooperate.^[113–115]^

Thirdly, authors within specific clusters exhibit less collaboration and fewer articles. They should consider forming collaborative groups. These clusters include altruistic punishment (#2), sustainable consumption dilemma (#8), structural solution (#10), and intergroup conflict (#11). For example, Ostrom E has the highest number of publications on altruistic punishment (#2), but most of his works are individually authored. Furthermore, other articles in the same cluster have different coauthors for each publication. This implies that outcomes in these research areas are relatively limited and new, requiring further exploration and development by additional researchers.

The conclusions drawn from the above analysis are as follows: (1) the analysis suggests that authors tend to produce more articles when they collaborate more. Collaboration with other researchers can significantly enhance the quality and quantity of research output. Therefore, we encourage researchers to seek collaborators who actively share their research interests. (2) Researchers in different disciplines exhibit distinct research focuses. Social statistical physicists prioritize the exploration of network reciprocity in social dilemmas, investigating the impact of group characteristics (such as size and interaction style) on people’s behavior. Conversely, social science researchers concentrate more on studying social dilemmas from the perspective of individual motivation and organizational behavior. (3) Researchers from diverse disciplines employ varying research methods. Statistical physicists predominantly utilize simulation experiments and modeling techniques to examine network reciprocity in social dilemmas, whereas social scientists often rely on laboratory experiments to conduct their research.

### 3.3. Co-citation analysis

This sector aims to answer Question 3 by co-citation analysis, primarily interpreting the relevant associations among authors, journals, and literature. The more frequently one author, Journal, or literature is co-cited, the stronger the impact of social dilemmas. Previous studies have shown that co-citation analysis enables us to comprehend the research status and evolution of an academic field.^[116]^ While some co-cited authors or references may not be included in our database, highly cited papers and authors are fundamental and pioneering in this area of research.^[117]^

#### 3.3.1. Journal co-citation

Table 6 exhibits the top 13 most co-cited journals in the social dilemma research domain. There are several findings. Firstly, journals with higher citation counts also exhibit higher impact factors, exemplified by Science (counts 1948, impact factor 54.5) and Nature (counts 1487, impact factor 60.9). Nine journals with impact factors exceeding 6 indicate a substantial influence on social dilemma research. Secondly, the centrality of journals varies from 0.00 to 0.07, indicating that the impact is different in this research domain. With a high centrality of 0.07, Science plays a crucial role in connecting other journals within the co-citation network. Thirdly, the cited journals span 5 disciplines: multidisciplinary sciences (Science and Nature), Political Science (The Journal of Conflict Resolution and American Political Science Review), psychology (Journal of Personality and Social Psychology and Annual Review of Psychology), economics (American Economic Review and Journal of Economic Behavior & Organization), and biology (Journal of Theoretical Biology). The diversity of disciplines implies that studying social dilemmas requires collaboration and contributions from multiple academic fields.

**Table 6:** Journal co-citation.

Ranking	Journals	5-year impact factor	Counts	Centrality	Year
1	Science	54.5	1948	0.07	1993
2	Nature	60.9	1487	0.03	1998
3	Proceedings of the National Academy of Sciences USA	12	1451	0.03	1997
4	Journal of Personality and Social Psychology	9.2	1373	0.01	1993
5	The American Economic Review	12.7	1242	0.03	1993
6	Journal of Theoretical Biology	2	945	0.02	1995
7	Annual Review of Psychology	29	913	0.01	1993
8	Journal of Economic Behavior & Organization	2.4	892	0.02	1996
9	PloS One	3.8	845	0.01	2008
10	Journal of Conflict Resolution	4.1	826	0.01	1993
11	Organizational Behavior and Human Decision Processes	6	810	0.01	1993
12	American Political Science Review	8.5	788	0.03	1993

**Figure 6.**
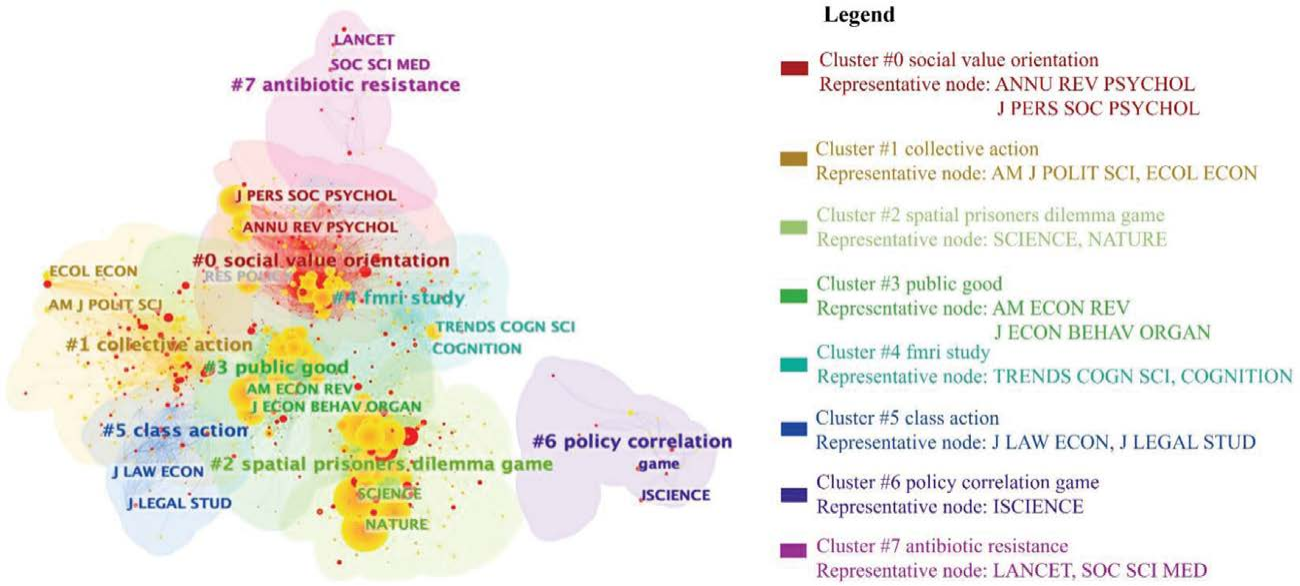
Journal co-citation clusters (nodes = 1151, links = 10,644).

Figure 6 shows the visualization of a journal co-citation network with 1151 nodes and 10,644 connecting lines, forming 7 clusters. From the visualization, 2 key insights emerge for researchers.

Firstly, researchers can strategically choose journals based on clusters. For example, those studying social value orientation should focus on psychology journals in cluster #0, such as the Journal of Personality and Social Psychology (counts 1373) and the Annual Review of Psychology (counts 913). Researchers exploring collective action (Cluster #1) may follow American Political Science Review (counts 273) and Ecological Economics (counts 275). Those interested in spatial prisoner dilemma games (#2) might consider Science (counts 1948) and Nature (counts 1487).

Secondly, journal citation counts vary significantly across different clusters. Specific journals in cluster #0 (social value orientation), cluster #2 (spatial prisoner’s dilemma game), and cluster #3 (public good) have citations exceeding 800, notably higher than other clusters. These highly co-cited journals provide valuable references for future studies in social dilemmas. Therefore, researchers can gain insights for selecting journals with high citation potential. Additionally, focusing on less active clusters could spur further exploration in specific domains of social dilemmas.

#### 3.3.2. Reference co-citation

Table 7 presents the top 13 co-cited references (cited 50 times or more) in the social dilemmas research domain. We can categorize these references into 3 types. (1) Reviews: Three reviews about cooperation evolutionary dynamics are produced from a physics perspective. One article reviewed how interactions between individuals and groups impact cooperative behaviors in social dilemmas.^[118]^ Another article summarized how different network structures alter and enrich long-term behavioral patterns in the prisoner’s dilemma.^[119]^ The third article overviewed universal scaling for the dilemma strength in evolutionary games.^[120]^ (2) Cooperative evolutionary mechanisms: Two reviews summarized cooperative evolutionary mechanisms such as direct reciprocity, indirect reciprocity, spatial selection, multilevel selection, and kin selection.^[5,121]^ (3) Factors influencing cooperation: The third category focuses on studying factors that foster cooperation among groups or individuals in social dilemmas, including group member heterogeneity, anonymity, and social diversity.^[122–124]^

**Table 7:** Reference co-citation.

Ranking	Reference	Counts	Centrality	Year
1	Perc M., Physics Reports, v687, p1	146	0.05	2017
2	Perc M., Biosystems, v99, p109	107	0.02	2010
3	Perc M., Journal of the Royal Society Interface, v10, p0	105	0.05	2013
4	Szabo G., Physics Reports, v446, p97	91	0.02	2007
5	Van Lange PAM., Organizational Behavior and Human Decision Processes, v120, p125	75	0	2013
6	Wang Z., The European Physical Journal B, v88, p0	70	0.13	2015
7	Rand DG., Trends in Cognitive Sciences, v17, p413	68	0.05	2013
8	Wang Z, Physics of Life Reviews, v14, p1	67	0.02	2015
9	Santos FC., Nature, v454, p213	63	0.01	2008
10	Roca CP., Physics of Life Reviews, v6, p208	63	0.02	2009
11	Santos FC., Proceedings of the National Academy of Sciences USA, v103, p3490	60	0.01	2006
12	Nowak MA., Science, v314, p1560	54	0.01	2006
13	Wang Z., Science Advances, v3, p0	51	0.01	2017

Among these authors, Wang Z contributed an article (2015) with a centrality value of 0.13. Additionally, Perc M (2017, 2013) and Rand DG (2013) published 3 articles with a centrality of 0.05. These co-cited articles with high centrality have expanded the research directions and content for subsequent authors. In exploring the factors influencing the evolution of cooperative behavior, subsequent authors have examined the effects of leaders’ behavior, third-party punishment, and seasonal variations on cooperation within social dilemmas.^[84,125]^ Additionally, other authors have investigated the mechanisms of human-machine cooperation and memory strategies within direct reciprocity.^[75,96,126]^

**Figure 7.**
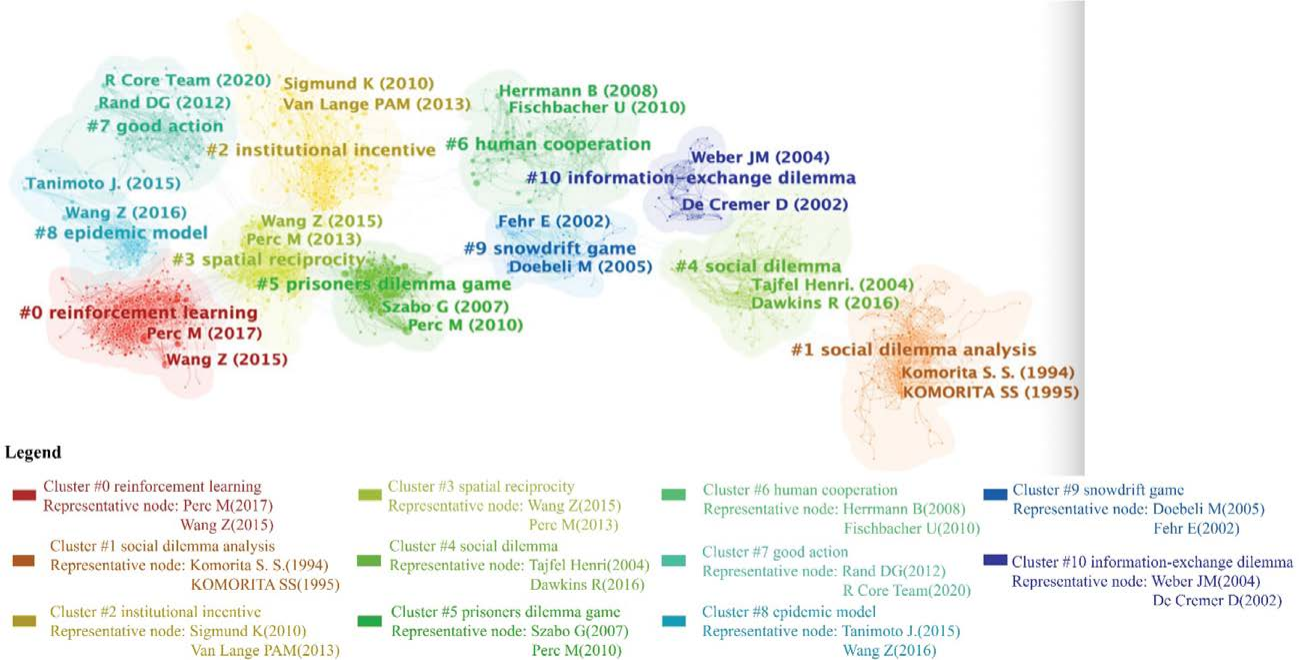
Reference co-citation clusters (nodes = 1652, links = 7507).

Figure 7 illustrates a visualization of the reference co-citation network, including the top 11 clusters (more than 20 articles), which helps us explore the subject distributions of the social dilemma research domain. These clusters can be further classified into 3 main groups: conditions for the evolution of cooperation in social dilemmas (clusters #0, #2, #3, and #7), social dilemma-related concepts (clusters #1, #4, and #6), and social dilemmas game paradigms (clusters #5, #8, #9, and #10).

Firstly, conditions for the evolution of cooperation in social dilemmas: The co-cited references related to this category primarily span from 2009 to 2020.

(1) Reinforcement learning (#0): This research theme has emerged as a recent research hotspot, including references exploring the role of rewards and punishments in addressing social dilemma problems. A highly cited reference by Perc M summarizes physics simulation experimental methodology and explores peer punishment and reward strategies to promote cooperation.^[39]^ Other co-cited references in this category focus on factors impacting cooperative behavior, including network structure, rewards (decoy effect), and social norms.^[127–129]^ One citing article recently expanded strategic punishment models, employing a switching strategy model to demonstrate that punishing the majority of betrayers promotes intragroup cooperation.^[38]^ Another citing article discovered that social punishment enhances cooperative behavior in multiplayer games.^[130]^
(2) Institutional incentive (#2): This clustering theme examines incentive measures to encourage cooperative behavior within organizations. The highly co-cited reference by Rand DJ in Science suggests that rewards are more effective than punishments in promoting cooperation. It proposes that human cooperation is supported by positive interactions with others.^[91]^ Another co-cited reference published in “Nature” points out that social learning contributes to forming institutions for managing public resources. Institutions formed in this way, such as collective punishment, can spontaneously emerge after multiple gaming rounds among organization members.^[131]^ Recent citing articles investigate the optimal incentive strategies for institutions. One article explored how the source of third-party punishment systems (endogenous or exogenous) affects their effectiveness, concluding that exogenous punishment systems are more effective.^[132]^ Another article found that the optimal punishing strategy is a more cost-effective way to achieve an expected cooperation level compared to the optimal rewarding strategy.^[133]^
(3) Spatial reciprocity (#3): Highly co-cited reference in this cluster explores whether group structure facilitates cooperation under social dilemmas, focusing on the role of group interactions in special public goods games.^[118,134,135]^ A recent citing article built a new spatial prisoner’s dilemma game with more enhanced network reciprocity.^[136]^
(4) Good action (#7): Two references written by David G and Rand DJ, published in “Nature,” discuss the cooperation heuristic and find that intuitive thinking increases the odds of cooperation.^[137,138]^

Secondly, the group of social dilemma-related concepts includes social dilemma analysis (#1), social dilemmas (#4), and human cooperation (#6). References in this group were published between 1989 and 2010. The representative co-cited references in social dilemma research (#4) focus on describing and explaining people’s interaction patterns in social dilemma problems. For example, one article summarizes research on two-person bargaining in social dilemmas.^[139]^ Another article summarizes the behavioral approach of collective action rational choice theory and provides a theoretical foundation for subsequent research on social dilemmas.^[3]^ References in cluster #6 include some reviews, such as the role of reciprocity, culture, and communication in influencing cooperation.^[1,140,141]^

Thirdly, the research themes in the social dilemma task include the prisoner’s dilemma (#5), information exchange dilemma (#8), snowdrift game (#9), and vaccination game (#10) within the context of epidemic spread. These highly cited articles explore cooperation models and their influencing factors under various game scenarios.^[142–144]^

The conclusions drawn from the analysis above are as follows: (1) the trend in research methods demonstrates diversification, transitioning from initial field surveys and laboratory studies to incorporating mathematical and physical simulation experiments. Using statistical physics to explore social dilemmas has been a hot research domain. (2) The literature review, adopting various disciplinary perspectives, can assist researchers in generating novel insights and exploring new research directions. (3) The hot trends have shifted from describing and theoretically explaining cooperation behavior to identifying factors and methods that promote cooperation in the social dilemma research domain. The above analysis can help future researchers understand how topics have changed and evolved. Additionally, these references can be a vital knowledge source in future research. Scholars may refer to these core references to stimulate the research process.

#### 3.3.3. Author co-citation

Table 8 exhibits 21 co-cited authors, with co-citations exceeding 350 times. Among these authors, Ostrom E (905 citations) made the most critical contributions in the social dilemma area, followed by Nowak MA, Fehr E, and Robert A, with 881, 850, and 820 citations. High citation counts for an author indicate the individual’s significant influence and recognition within the social dilemmas research domain. Thus, these authors are the most influential researchers in this research domain. In particular, Ostrom E, the highest co-cited author, focusing on the “tragedy of the commons,” received the 2009 Nobel Prize in Economics for her work in sustainable resource management.^[145]^ This author’s essential book, “Governing the Commons: The Evolution of Institutions for Collective Action,” has substantially impacted social dilemma research. Nowak MA proposed that cooperation, the third principle of human evolution, is crucial for a group’s survival.^[124]^ Fehr E developed the inequity aversion model, incorporating a fairness preference on the traditional self-interest assumption to explain people’s economic behavior.^[146]^ Axelrod R. is the author of “The Evolution of Cooperation.” This author holds the highest centrality (0.16) among the top 22 co-cited authors, indicating this author’s significant impact on social dilemma research.^[147]^ In addition, comparing the author collaboration network and the author co-citation network, we find that the most prolific authors are also highly co-cited, consistent with other previous studies.^[116]^

**Table 8:** Author co-citation.

Ranking	Author	Counts	Centrality	Year
1	Ostrom E	905	0.04	1993
2	Nowak MA	881	0.07	2003
3	Fehr E	850	0.03	2000
4	Axelrod R	820	0.16	1993
5	Dawes RM	778	0	1993
6	Hardin G	681	0.05	1993
7	Perc M	489	0.04	2009
8	Flschbacher U	483	0.02	2004
9	Santos FC	482	0.02	2007
10	Szabo G	480	0.02	2008
11	Szolnoki A	473	0.02	2009
12	Yamagishi T	470	0.05	1993
13	Hauert C	461	0.02	2005
14	Van Lange PAM	427	0.02	1996
15	Messick DM	420	0.02	1993
16	Kollock P	395	0.02	2001
17	Rapoport A	390	0.02	1993
18	Rand DG	375	0.05	2011
19	Milinski M	369	0.02	2004
20	Andreoni J	369	0.03	1996
21	Wang Z	353	0.03	2012

**Figure 8.**
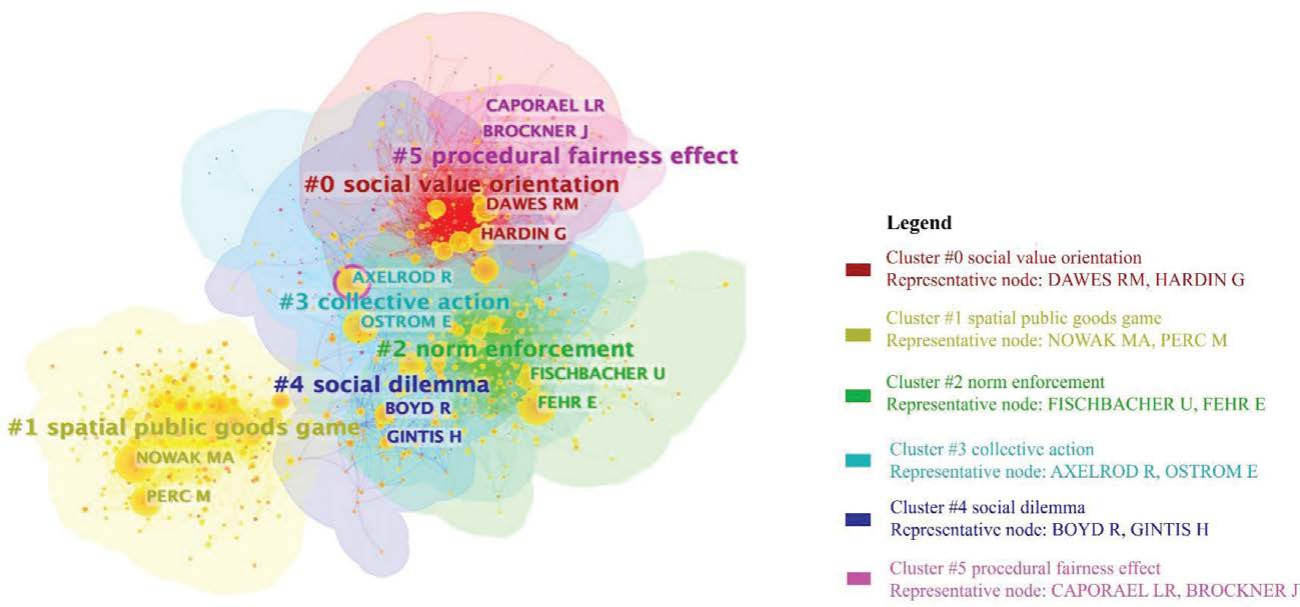
Author co-citation clusters (nodes = 1132, links = 10,610).

Figure 8 shows a visualization of the author’s co-citation network and summarizes valuable information about the research topic. There are 6 main category clusters.

Social value orientation (#0): This cluster focuses on individuals’ self-interest considerations versus others, such as selfishness or altruism. Dawes RM (778 co-citations) and Hardin G (681 co-citations) are often co-cited heavily in this area. Recent researchers found significant effects of social value orientation on the willingness to share information, support pro-nuclear policies, and resolve group conflicts.^[148,149]^

Public goods game (#1): Nowak MA (881 co-citations) and Perc M (489 co-citations) are prominently cited in this area. Nowak MA was the first to identify spatial reciprocity in spatial prisoner’s dilemma game tasks,^[150]^ while Perc M has contributed significantly to spatial reciprocity using statistical physics methods.^[34]^ Based on these references, recent citing articles explored the impact of investment allocation schemes and synergistic discounting on evolutionary cooperation in the spatial public goods game.^[151,152]^

Norm enforcement (#2): This cluster investigates the role of norm enforcement (often through punishment) in resolving collective action dilemmas. Prominent authors in this area include Fehr E (850 co-citations) and Flschbacher U (483 co-citations). Recent co-cited articles highlight the significance of pro-social punishers and tight informational networks among group members for initiating and sustaining cooperation.^[153,154]^

Collective action (#3): Axelrod R (820 co-citations) and Ostrom E (905 co-citations) are prominent authors in this field. Their insights in addressing collective action problems have found application in densely populated agricultural landscapes and environmental disaster reduction.^[155,156]^

Social dilemmas (#4): Boyd R (302 co-citations) and Gintis H (207 co-citations) are frequently co-cited in this field. They focused on altruistic punishment, strong altruistic reciprocity, and cooperation. Their studies have aided recent researchers in investigating peer punishment, leadership, and the role of fair punishment in solving social dilemmas.^[157,158]^

Procedural fairness effect (#5): Caporael LR (27 co-citations) and Brockner J (21 co-citations) are primary co-cited authors. Recent co-cited articles found that procedural fairness facilitates cooperative behavior by enhancing cooperative expectations.^[159,160]^

We can obtain 3 conclusions from the above analysis. (1) Based on bounded rationality, Ostrom E. proposed that organizations and individuals solve social dilemmas through self-organization and self-adaptation. This method provided a theoretical basis for subsequent social physicists such as Perc M. (2) Value orientation, norms, and fairness are the critical factors influencing people’s behavior choices in social dilemmas. Researchers pay much attention to them. (3) The emergence of spatial public goods games as the newest co-citation topic indicates that it is currently a hot research direction. These results provide insights into whom to track when scholars seek to identify the most influential scholars and quickly understand the evolution of research topics within the social dilemma research domain.

### 3.4. Co-occurrence analysis

This sector aims to answer Question 4 by co-occurrence analysis focusing on frequent categories and keywords in the literature to reveal their relationships. The aim is to showcase the themes of the literature, construct relationships between studies, understand past research hotspots, and speculate on potential future research directions.^[161]^

#### 3.4.1. Category co-occurrence

Table 9 presents the top 17 co-occurrences of categories with over 100 articles in social dilemma research. The top categories are mainly related to psychology, economics, and physics. In detail, the most important category is psychology (798), including social psychology, multidisciplinary psychology, and applied psychology, with counts of 398, 251, and 143, respectively. Economics (562) ranks second, followed by physics (427), multidisciplinary science (257), and political science (290). Besides, economics has a high centrality of 0.32, followed by environmental studies (0.26) and psychology multidisciplinary (0.19), indicating these categories play vital roles in connecting other categories in the social dilemma research co-occurrence network.

**Table 9:** Category co-occurrence.

Ranking	Category	Counts	Centrality	Year
1	Economics	562	0.32	1993
2	Social Psychology	398	0.02	1993
3	Multidisciplinary Science	317	0.11	1994
4	Political Science	290	0.02	1993
5	Multidisciplinary Physics	257	0	1999
6	Multidisciplinary Psychology	251	0.18	1993
7	Environmental Studies	243	0.26	1993
8	Management	203	0.08	1994
9	Mathematical Physics	170	0.02	2002
10	Sociology	161	0.05	1993
11	Applied Psychology	143	0.04	1993
12	Biology	143	0.07	2005
13	Environmental Sciences	138	0.14	1996
14	International Relations	133	0.01	1993
15	Mathematics, Interdisciplinary Applications	130	0.19	1993
16	Mathematical & Computational Biology	113	0.02	1993
17	Ecology	107	0.18	1993

**Figure 9.**
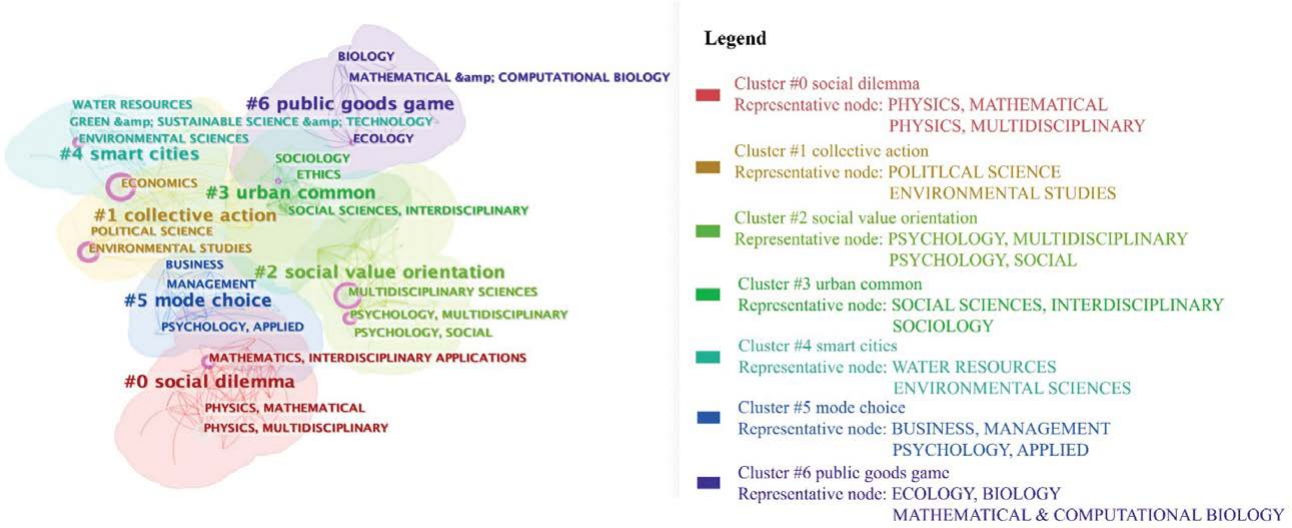
Category co-occurrence clusters (nodes = 161, links = 611).

The cluster view of the category co-occurrence is shown in Figure 9, which helps us explore the subject distributions of this domain. There are 7 clusters: social dilemma, collective action, urban public space, smart city, decision-making, and public goods gaming.

Social dilemma (#0): This cluster contains multidisciplinary physics, physical mathematics, and interdisciplinary applications of mathematics, with counts of 275, 170, and 130. Researchers focus on the evolution of cooperation in social dilemmas; they explore individual and environmental factors influencing cooperation in social dilemmas, including aspects such as reputation, unequal dependence on group members, crowdfunding mechanisms, and reinforcement learning.^[162–165]^

Collective action (#1): This cluster includes economics, political science, and environmental studies, with the counts of 562, 290, and 243. Researchers explore the impact factors and solutions of social and economic problems, such as the housing cooperative for low-income families, house management system, waste separation, and large-scale collective action of COVID-19.^[166–169]^

Social value orientation (#2): This cluster includes social psychology, multidisciplinary science, and multidisciplinary psychology, with counts of 398, 317, and 251. Researchers primarily explore human cooperation’s psychological and brain mechanisms through observation and experiments. For instance, some researchers study experiential learning, deliberation, and perceived social consensus in cooperative decision-making.^[170–172]^ Others employ event-related potential techniques to identify brain region activity, offering evidence that motivation and behavioral relevance are the neural foundations of cooperation.^[173]^

Urban common (#3): This cluster encompasses sociology, social science, and ecology, with counts of 161, 71, and 51, respectively. Researchers pay attention to social dilemmas in the medical field, examining aspects such as the mental health of chronic patients, antibiotic use, the utilization of reproductive technologies, and gender changes.^[173–175]^ Additionally, they investigate solutions at the legal, ethical, and social consensus levels.^[176–178]^

Smart cities (#4): This cluster contains water resources science, green sustainable disciplines, and environmental science. Researchers focus on social dilemma issues associated with urban and natural environments, including the conflict between agricultural production and environmental protection, the dilemma of smart city development, cooperation in grain storage, and climate change. They seek to address these challenges by establishing trust mechanisms (among citizens, between the government and citizens), optimizing the organizational forms of social institutions, and facilitating process control.^[179–181]^

Mode choice (#5) This cluster includes management, applied psychology, and business disciplines. Researchers primarily focus on social dilemma issues in organizational and management domains, such as knowledge sharing in organizations and lane-changing vehicles in traffic management. They study how to increase cooperation in these areas through enhancing goal orientation, improving communication, and leveraging leadership.^[176,182,183]^

Public goods games (#6) encompass biology, mathematics, and ecology disciplines. In recent years, researchers have focused on the evolutionary dynamics of cooperation, utilizing simulation experiments to study the influence of reward variability on cooperation in social dilemmas.^[184,185]^ These studies reveal the evolutionary dynamics of cooperation.^[186,187]^

Based on the above analysis, specific directions and emphases on social dilemmas vary across these disciplines. Social physics and mathematics focus on the evolutionary dynamics of cooperation. Psychology prefers to study the behavioral motivations of individuals in social dilemmas, such as social norms and values. Economics, political science, and environmental studies address social dilemmas such as household management systems, waste separation, and climate change. Researchers can explore relevant studies in different disciplinary fields based on their research focus, thereby gaining new perspectives.

#### 3.4.2. Keywords co-occurrence

The keyword co-occurrence can help researchers understand the evolvement of keywords and hot topics in social dilemma research.^[188]^ We conducted an in-depth keywords co-occurrence analysis. Table 10 lists the top 21 keywords co-occurrence with counts of articles exceeding 150. The more counts of keywords appear, the hotter the keywords. These keywords have been mentioned previously in this paper, and we can find 3 hot topics in detail. (1) Critical concepts include keywords of “social dilemma” (counts 1499), “cooperation” (counts 939), “evolution” (counts 410), and “collective action” (counts 362). (2) Social dilemma tasks include “prisoner’s dilemma” (counts 466), “public goods” (counts 428), and “evolutionary games” (counts 229). (3) Factors influencing cooperation in social dilemmas include “punishment” (counts 234), “strategy” (counts 170), “trust” (counts 158), “fairness” (counts 148), and “group size” (counts 146). Among them, 7 keywords’ centrality exceeds 0.05, including “social dilemma,” “cooperation,” “prisoner’s dilemma,” “collective action,” “decision-making,” “altruism,” and “group size.” The high centrality indicates that these keywords are crucial in connecting different keywords and hold a dominant position in this research domain.

**Table 10:** Keyword co-occurrence.

Ranking	Keyword	Counts	Centrality	Year
1	social dilemma	1499	0.06	1993
2	cooperation	939	0.05	1993
3	prisoners dilemma	466	0.05	1993
4	public good	428	0.03	1993
5	evolution	410	0.03	1993
6	collective action	362	0.05	1993
7	game	356	0.02	1993
8	dynamics	303	0.02	2005
9	model	262	0.04	1993
10	reciprocity	257	0.03	2001
11	punishment	234	0.01	2002
12	decision making	234	0.07	1993
13	evolutionary game	229	0.02	2007
14	communication	171	0.04	1994
15	strategy	170	0.04	1996
16	trust	158	0.01	1999
17	public goods game	155	0.01	2004
18	networks	149	0.02	1993
19	fairness	148	0.02	1997
20	altruism	146	0.05	1994
21	group size	146	0.07	1993

**Figure 10.**
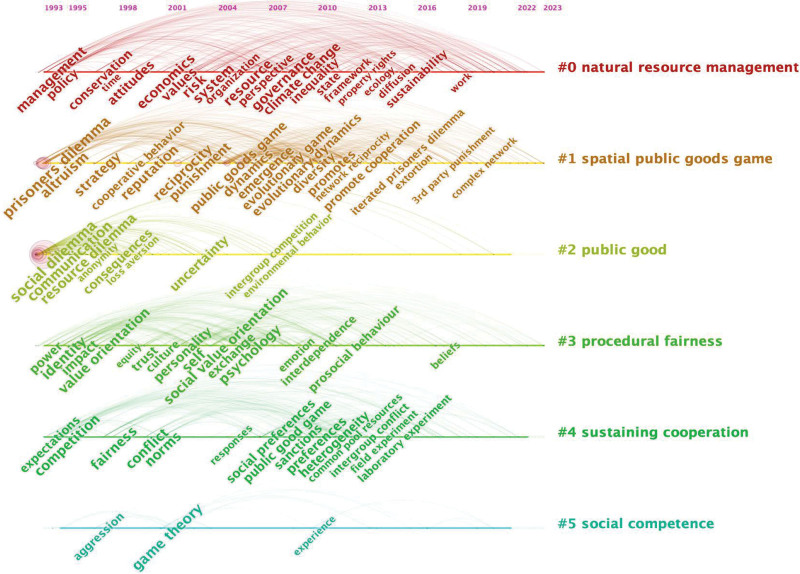
Timeline of keyword co-occurrence (nodes = 885, links = 7893).

Figure 10 shows the timeline of keyword co-occurrence, which consists of 894 nodes and 7994 links. We obtained 7 clusters: natural resource management (#0), Spatial public goods game (#1), public good (#2), procedural fairness (#3), sustaining cooperation (#4), cooperation psychological game theory (#5), and resource provision (#6). We can observe the evolution of hot topics and keywords by analyzing the temporal dynamics and the counts of the keywords in the social dilemma research domain. The hot topics are as follows:

(1) Natural resource management and global climate change governance represent critical research directions within cluster #0. During 1993 to 2003, co-occurrence keywords included “management,” “policy,” “protection,” “attitudes,” and “risks.”^[189–192]^ During 2004 to 2013, the co-occurrence keywords covered “resources,” “climate change,” “inequality,” and “property rights.”^[193–195]^ During 2014 to 2023, co-occurrence keywords focused on “sustainability” and “policy network issues.”^[196–199]^
(2) The study of human behavioral tendencies (cooperation or betrayal) and the relevant influencing factors in social dilemmas has garnered significant attention from researchers. Relevant co-occurrence keywords are explored in both #1 and #2. #1 Spatial public goods game delves into the evolutionary mechanisms of cooperation in social dilemmas. During 1993 to 2003, co-occurrence keywords include “evolution,” “altruism,” “strategy,” and “reputation.” Some studies have challenged the traditional assumption of rationality in economic agents by demonstrating people’s capability for cooperation in social dilemmas.^[200,201]^ During 2004 to 2013, co-occurrence keywords include “punishment,” “public goods game,” “dynamics,” “diversity,” “evolutionary dynamics,” and “promoting cooperation.” Researchers used evolutionary game theory to illustrate cooperative behavior in the traveler’s dilemma and the centipede game.^[202–204]^ During 2014 to 2023, researchers focused on “third-party punishment” and “complex network cooperation.” They found some negative impacts of punishment and social norms on cooperation.^[205,206]^

#2 Public good encompasses critical early and prominent keywords of the evolution of cooperation in social dilemmas, including “social dilemma,” “resource dilemma,” and “communication.” These keywords appeared in 1993. The “social dilemma” is directly related to the topic of this paper and appears most frequently. Researchers sought the group size, democracy, economic uncertainty, number of players, communication, and other factors that impact people’s cooperation in social dilemmas.^[207–209]^

The above analysis shows that the public goods dilemma serves as the null model for human behavior in social dilemmas, extending toward factors such as punishment, reward, group size, and trust. Future researchers can utilize these keywords to comprehend the crucial situational factors influencing cooperative behavior in social dilemmas.

(3) Other factors influencing people’s behavior in social dilemmas are their psychological feelings and individual differences. These include the sense of fairness, emotions, trust, and social value orientation. Fairness and the related factors influencing people’s cooperation are popular topics in social dilemma research. There are more related co-occurrence keywords in “#3 procedural fairness.” During 1993 to 2003, co-occurrence keywords included “identity,” “trust,” and “value orientation.” Researchers have shown particular interest in understanding people’s cooperative preferences when they perceive procedural fairness and charismatic leadership.^[210,211]^ During 2004 to 2013, co-occurring keywords expanded to include “social value orientation,” “emotions,” and “personality.” Researchers focus on the relationship among procedure fairness, social value orientation, emotions, and trust. Their findings indicate that individuals who perceive fairness and trust in developing rules are more inclined to cooperate.^[212,213]^ During 2014 to 2023, co-occurrence keywords extended to include “prosocial behavior” and “beliefs.” Recently, researchers studied the prosocial behavior of wearing a mask to prevent the spread of diseases underpinned by evolutionary game theory.^[214,215]^ Another study revealed that pessimistic beliefs about others’ contributions are associated with corruption in heterogeneous groups, negatively affecting cooperation.^[216]^ These keywords can help researchers understand the individual factors that influence people’s behavior choices in social dilemmas. The primary researchers conducting these studies are social scientists, particularly psychologists and organizational behaviorists.
(4) Sustaining cooperation (#4) seeks factors influencing people’s sustaining cooperation behavior in various social dilemmas. During the period from 1993 to 2003, researchers focused on examining the influence of keywords such as “competition,” “fairness,” and “norms” on sustaining cooperation in social dilemmas.^[201,217,218]^ During 2004 to 2013, co-occurrence keywords included “social preferences, “public good game, and “preference.”^[140,219,220]^ During 2014 to 2023, co-occurrence keywords include “intragroup conflicts, “laboratory experiments, and “field experiment.”^[221–223]^ From these analyses, the factors affecting sustained cooperation mainly include group conflict and individual behavioral preferences.
(5) Social competence (#5) has fewer co-occurrence keywords. It means that research within these clusters is more specific and detailed, receiving less sustained and extensive attention than other clusters. Concerning “game theory,” researchers have developed a theoretical model to explain builders’ low acceptance of green building practices.^[224]^ As for “aggression,” some researchers have explored the social dilemmas caused by aggression, such as intergroup conflicts. They investigate the effects of spoils, division rules, and group reputation on aggression.^[225–227]^

In conclusion, 3 critical suggestions for researchers emerge from the above analysis. Firstly, co-occurring keywords and emerging trends constantly change, meaning many new hot topics have emerged. Therefore, keeping an up-to-date understanding and competitive predominance in this research domain is necessary. Secondly, some research topics consistently draw researchers’ attention and evolve. Notably, the keyword “punishment” has transformed into more nuanced concepts such as “third-party punishment” and “peer punishment.” We suggest that researchers explore the evolutionary trend of these hot topics and seek new research directions accordingly. This result can help new research entrants quickly comprehend this research domain’s fundamental concepts and hot topics in different periods.

#### 3.4.3. Keywords bursts

**Figure 11.**
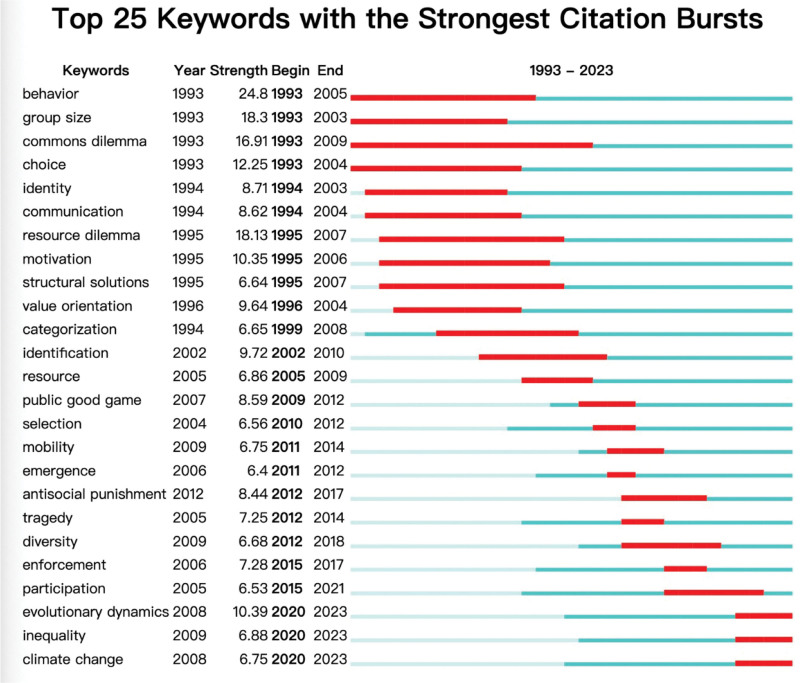
Top 25 keywords with the strongest citation bursts.

The keyword bursts reflect the research frontier status of social dilemmas through the dynamic changes of keyword citation.^[228]^ Figure 11 presents the top 25 keywords with the strongest citation bursts from 1993 to 2023. We can observe that the frontier topics of social dilemmas have changed over time. Here are some findings:

(1) The top 4 keywords with the strongest, longest, and earliest bursts are “behavior” (strength 24.8), “group size” (strength 18.3), “commons dilemma” (strength 16.91), and “choice” (strength 12.25). It means these keywords are frontier topics within their corresponding period and have foundational significance in social dilemmas demand. Researchers have focused on the effect of group size on people’s behavioral choices in social dilemmas (cooperation or betrayal). Earlier studies explored whether members of large or small groups were more cooperative and found that small groups would promote cooperation. Recently, researchers have found that we can address collective-risk social dilemmas through mechanism design by forming these smaller groups.^[208,229]^ Researchers have also found that when the cost of cooperation is low enough, the probability of cooperation increases with group size.^[230]^
(2) There are another 5 keywords that have been exploding for >10 years, including “communication” (11 years), “resource dilemma” (13 years), “motivation” (12 years), “structured solution” (13 years), and “categorization” (10 years). They are mainly focused keywords and are likely to be the potential turning points for hot topics in social dilemmas. Resource dilemma is a basic prototype of the social dilemmas, in which an aggregate of people shares a slowly replenishing “resource pool” out of which each person can “harvest” a significant amount at any time. Regarding resource dilemma research, the researchers found that management systems, leaders, and a sense of fairness all influence the level of cooperation among individuals in resource dilemmas.^[47,231]^ Communication is well-known to increase cooperation rates in social dilemmas. The researchers followed how different modes of communication influence the cooperation of individuals.^[232–234]^
(3) The up-to-date top 3 keywords are “evolutionary dynamics,” “climate change,” and “inequality.” These keywords are the latest research frontier topics of social dilemma demand. Among them, the “evolutionary dynamics” also have high strength, indicating that this topic is a hotpot research direction. “Evolutionary dynamics” focuses on how individuals adjust their cooperation strategies in different environments. Researchers are mainly focused on the dynamic roles of fairness, reciprocity, reward, and punishment in the evolution of cooperation.^[235–239]^ The “climate change” research seeks various methods to promote environmental protection. Researchers utilize social norms, reputation, and adjustments in psychological distance to raise individuals’ concerns about climate change.^[240–242]^ Research on “inequality” delves into the impact of misunderstandings, trust issues, and unequal opportunities (including disparate access to resources) on pro-environmental behavior.^[243–245]^

To summarize, several frontier topics emerge in current social dilemma research. (1) Resource dilemma and climate change remain the primary focuses of study within social dilemmas. (2) Inequality, reciprocity, reward, and punishment are significant factors influencing the evolution of cooperative behavior. However, recent studies suggest these factors may interact differently under various environmental conditions. (3) The influence of group size continues to be a significant area of study, with ongoing research exploring the conditions that promote cooperation across varying group sizes. These findings enable new scholars to quickly grasp the current frontiers, reduce potential research costs, and enhance their research efficiency when initiating their studies.

## 4. Discussions

### 4.1. Knowledge framework

**Figure 12.**
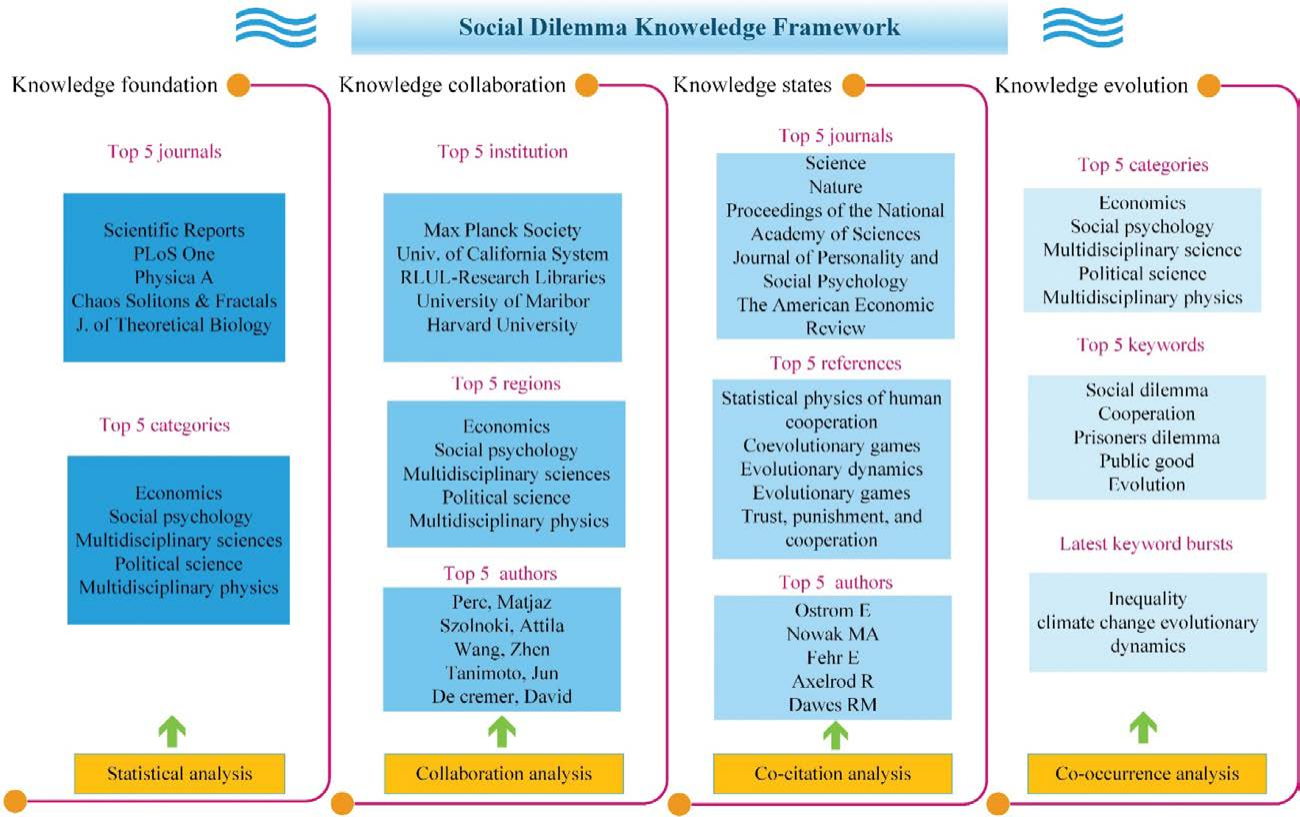
Knowledge framework. (*Note*: Top 5 means that the counts of research articles in journals, institutions, categories, regions, and authors et al within this research domain are among the highest 5.)

Previous research reviews on social dilemmas have often overlooked the interdisciplinary knowledge framework. By establishing an interdisciplinary knowledge framework, we can comprehensively understand the current research status, identify research hotspots, and anticipate future trends in the field.^[246]^ Therefore, we have established the following knowledge framework. This framework encompasses 4 main pillars: knowledge foundation, knowledge cooperation, knowledge status, and knowledge evolution. By integrating insights from multiple disciplines, such as psychology, sociology, and economics, this framework offers a holistic understanding of the current state of social dilemmas. It also highlights research trends within this domain, facilitating a more intuitive depiction of the field (Fig. 12).

In the knowledge foundation section, statistical analyses of categories and publications are conducted in the domain of social dilemma research. We have found that this research domain is multidisciplinary and hot. Economics, psychology, political science, interdisciplinary science, and physics are the most focused disciplines. Scientific Reports, PLoS One, Physica A: Statistical Mechanics and its Applications, Chaos, Solitons & Fractals, and Journal of Theoretical Biology are the journals publishing most papers in this research domain. Different disciplines integrate their perspectives and methods to study and solve social dilemmas at various levels. Economists examine the effects of incentives, penalties, and fairness on social dilemmas. They propose economic models and design relevant policies and institutions from the governmental and organizational levels. Psychologists use experiments and surveys to understand how cognition, motivation, and social norms affect behavioral choices in social dilemmas, offering recommendations for individual behavioral interventions. Sociologists focus on social structures and group dynamics, analyzing the role of cultural values in social dilemmas to clarify the social context of personal behavior. Sociophysicists and computational social scientists use physical simulations, computer models, and big data to simulate the dynamic changes in individual and group behavior and explore the evolution of behavior in social dilemmas.

In the knowledge collaboration section, the institutions, regions, and authors that collaborate more frequently are found by collaboration analysis. The Max Planck Institute for Human Development, the University of California, and the RLUL-Research Libraries are the more collaborative institutions in the social dilemma research domain. The United States, China, Germany, and the United Kingdom are the major regions that have initiated cooperation. Perc M, Szolnoki A, and Wang are the authors with the most active collaboration. Collaboration among institutions, regions, and authors from different disciplines integrates theories, methods, and data to propose comprehensive solutions. For example, in addressing climate change and public health crises, economists can design policies, psychologists can suggest individual behavior interventions, sociologists can explore strategies effective in different cultural contexts, and environmental scientists can propose sustainable development solutions based on resource management.

In the knowledge states section, we can know the current research state and themes from the analysis of co-cited journals, references, and authors. This analysis shows that co-cited journals and authors focus on social value orientation, spatial prisoner dilemma games, and public good. These themes are the most crucial topic in this research domain. For example, Social Value Orientation is a foundational theory with significant citations in this field. Different Social Value Orientation types: such as individualism, cooperation, altruism, and competition: affect individuals’ decisions about resource allocation, fairness judgments, and cooperative behaviors. This theory helps researchers predict individual behavioral choices, understand the motivations behind cooperation and conflict in social dilemmas, design targeted behavioral interventions, and explain cross-cultural behavioral differences. Besides, reinforcement learning, institutional incentives, spatial reciprocity, and good action are the prevalent themes in co-cited journal analysis results. Norm enforcement and procedural fairness are the hot themes in the co-cited author’s analysis results.

In the knowledge evolution section, the research hot and frontier topics from category and keyword occurrence. The co-occurring categories are the same as those in the knowledge base section. Keyword co-occurrence and burst analysis depict the evolution of the research topic and future trends in the social dilemma research domain. Climate change, evolutionary dynamics, and inequality reflect research frontiers and trends in this domain. For example, the evolutionary dynamics of individual behavior in social dilemmas have become a major research focus across various disciplines in recent years. Economists use game theory models to study how strategic choices and equilibrium analysis explain behavior in social dilemmas, examining conditions under which cooperation or defection evolves. Psychologists focus on cognitive and emotional processes, investigating how empathy, anger, and guilt drive decisions that promote cooperation or lead to conflict. Sociophysicists apply methods and models from physics to study the evolution of cooperative behaviors under different conditions. This multidisciplinary integration of economic theories, psychological insights, and physical models helps comprehensively understand the evolutionary dynamics of individual behavior in social dilemmas. This part can help scholars clarify the skeleton of knowledge development, understand the evolution of this domain’s topics, and grasp the research frontiers.

### 4.2. Key research directions

Analyzing knowledge foundations, collaboration, status, and evolution reveals that this domain has significant academic value, necessitating continuous scholarly exploration. Meanwhile, social dilemmas related to consumption, investment, social interactions, and environmental protection continuously emerge in contemporary society. Fostering cooperation in human social dilemmas requires a comprehensive theoretical and applicatory resolution. Therefore, future research needs to focus on 3 key aspects.

(1) Foster interdisciplinary collaboration

Future research efforts should foster collaboration among economists, psychologists, neuroscientists, biologists, physicists, and mathematicians. This interdisciplinary approach is essential because, in the face of social dilemmas such as the tragedy of the commons, it allows for the synthesis of research and the proposal of solutions from multiple perspectives. Economists use game theory and economic models to analyze the behavior of individuals and groups in resource sharing, designing incentives and policies to encourage sustainable resource management. Psychologists conduct experiments and surveys to study the effects of individual cognition and emotion on behavior in these dilemmas. They develop education and outreach strategies to increase public awareness of environmental protection, leveraging cognitive factors (like group identity) and emotional drivers (such as guilt and pride). Social physicists use physical simulations to model the proposals made by economists and psychologists, identifying effective policy measures to promote cooperative public behavior in social dilemmas. Computational social scientists analyze big data to develop refined management strategies for different groups, improving the implementation of relevant policy measures. Through comprehensive interdisciplinary research and collaboration, it is possible to integrate the strengths of different disciplines to form cohesive policy measures that promote sustainable social development.

(2) Establish new theoretical frameworks

The field of social dilemma research requires theoretical integration. Currently, theories on social dilemmas include instrumental rationality, bounded rationality, and social interaction theory. These theories offer different perspectives on understanding and addressing social dilemmas. Drawing from instrumental rationality, researchers have proposed theories such as kin selection, reciprocal altruism, and group selection to elucidate cooperative behavior. Additionally, they have suggested strategies such as reward and punishment to facilitate cooperation. On the other hand, bounded rationality-based studies propose solutions such as autonomous governance in policy research, heuristic decision rules in behavioral research, and spatial dynamics in exploring the influence of group structure.

However, these theories only partially explain human behavior and coping strategies in social dilemmas, sometimes leading to conflicting results. For instance, while some studies support the effectiveness of punishment strategies, others do not. Moreover, there is a lack of research based on bounded rationality and relevant solution strategies. Therefore, there is a need for theoretical integration grounded in bounded rationality to analyze the boundary conditions under which various strategies are effective. This integration can assist in determining when to utilize punishment strategies versus reward strategies, ultimately leading to improved solutions for real-world problems such as environmental pollution, conflicts, and competitive commercial practices.^[40,247,248]^

(3) Research methods need updating

Traditional methods of researching social dilemmas include biological experiments, social observations, mathematical and physical modeling, and psychological experiments. Quantitative studies, such as experiments and simulations, have been prevalent with less qualitative research. Laboratory experiments have been favored over studies in natural social settings due to their ability to control irrelevant variables, thus providing more accurate insights into individuals’ behavioral rules and influencing factors in social dilemmas. For example, social dilemma tasks like the Prisoner’s, public goods, and snowdrift dilemmas can simulate real-life situations and offer experimental control. However, concerns regarding their ecological validity persist. Consequently, researchers need to explore effective methods and leverage emerging technologies for studying social dilemmas in natural social settings.

Firstly, exploring virtual reality can facilitate experiments replicating real-world scenarios, such as business negotiations. Secondly, it is suggested to enhance qualitative analysis by adopting methodologies like Krippendorff Alpha, enabling researchers to assess the consistency and reliability of their qualitative analysis, mainly when dealing with subjective assessments or categorizations of text collected from social media.^[248]^ Thirdly, researchers can conduct more field studies to validate the findings of laboratory experiments, such as observing the impact of specific rewards or punishments on people’s donation behavior and environmental protection behavior. By incorporating these adjustments, researchers can aim for more coherent and logically structured studies on social dilemmas, thus advancing our understanding of these complex social dynamics.

(4) Explore social dilemmas in real-world contexts

Researchers have recently focused on protecting the environment, preserving public lands, understanding group cooperation and conflict, and managing diseases and vaccines. However, they have not paid as much attention to newer social problems caused by technology and social changes. Examples of these new problems include concerns about online privacy, fair distribution of resources during crises, income inequality, fair access to education, political divisions, and ethical shopping choices. For example, people often choose convenience over sustainable practices, which harms the environment. As society deals with these complicated issues, there is a growing need for different fields to work together and find new solutions.

## 5. Conclusions

### 5.1. Critical conclusions

This study represents the first attempt to conduct a bibliometric analysis of social dilemma research over the past 30 years to identify the knowledge framework. Through quantitative analysis of 3630 documents using CiteSpace software, we delineated the knowledge foundation, collaboration, status, and evolution within this research domain, presenting a comprehensive review. In the knowledge foundation section, statistical analyses of categories and publications reveal that this research domain is multidisciplinary and highly active. In the knowledge collaboration section, collaboration analysis identifies the institutions, countries, and authors that collaborate most frequently. In the knowledge states section, analysis of co-cited journals, references, and authors provides insight into the current research state and themes. In the knowledge evolution section, the analysis of category and keyword occurrence over time reveals research hotspots and frontier topics, showing their dynamic changes through time slices.

By analyzing these 4 aspects, we can identify the core research institutions, authors, and literature in this field, the main collaborating groups, current hotspots, and future trends. This comprehensive knowledge framework enables future scholars to understand this research field’s development context and frontier dynamics more thoroughly, allowing them to propose more innovative and practical research programs. Drawing upon these findings, we can answer the 4 questions in the introduction and derive the following critical conclusions:

(1) We completed the statistical analysis of publications, answering the Question 1 proposed in the introduction. Social dilemmas research is crucial, popular, and interdisciplinary. Publications indicate a fluctuating upward trend in social dilemma studies, with journals encompassing diverse categories. There is a growing necessity to encourage interdisciplinary collaboration in the future, aiming to offer a comprehensive understanding of the mystery of human cooperation in social dilemmas.
(2) We completed the analysis of collaboration among regions, institutions, and authors, addressing the Question 2 query proposed in the introduction. Collaboration in the social dilemma research domain has progressed among regions, authors, and journals. This collaboration is notable within specific disciplines, countries, and institutions, particularly in economically developed regions. Critical topics in institutional collaboration include investigating the evolutionary dynamics of human behavior, cooperation, and reinforcement learning. Additionally, Researchers from different disciplines demonstrate varying research focuses. Social statistical physicists prioritize exploring network reciprocity in social dilemmas, investigating the influence of group characteristics such as size and interaction style on human behavior. Conversely, social science researchers predominantly study social dilemmas from the perspective of individual motivation and organizational behavior. However, cooperation among regions, institutions, and authors tends to be limited to within disciplines and institutions. There is less of interdisciplinary, cross-institutional, and cross-national collaboration. To effectively tackle global social dilemmas like climate change, public health, and energy, governments can incentivize and finance transnational, interdisciplinary cooperation projects and establish joint laboratories or research centers. This integrated approach is crucial for generating comprehensive solutions to significant social challenges and fostering innovation and breakthroughs in these critical research areas.
(3) Based on the co-citation analysis of journals, references, and authors, we have addressed Question 3. The research themes and methodologies in social dilemmas have shown remarkable continuity over the past 30 years. However, there has been a shift in hot trends, moving from simply describing and theoretically explaining cooperation behavior to identifying factors and methods that promote cooperation within the social dilemma research domain. Factors such as value orientation, norms, and fairness play crucial roles in influencing people’s behavioral choices in social dilemmas, garnering significant attention from researchers. The emergence of spatial public goods games as the newest co-citation topic indicates a current hot research direction in the social dilemma.
(4) we addressed Question 4 and got the research hotpots and frontiers based on the keywords co-occurrence analysis. Current research hotpots include evolutionary dynamics, inequality, and climate change. Additionally, there is a growing emphasis on the impact of institutions, punishment, and rewards in promoting cooperation. These research hotspots provide directions for future researchers to delve into and address issues related to social dilemmas. Additionally, researchers should take note of the evolving of keywords to generate new research ideas.

### 5.2. Innovations, implications, and limitations

This study demonstrates innovation in several aspects. (1) We have established a comprehensive and precise knowledge framework for social dilemma research over the past 30 years. This framework will assist scholars new to the field gain a more comprehensive and dynamic understanding of the evolution of knowledge within this research domain. (2) We summarize the frontiers and trends within the social dilemma research domain, offering valuable references and identifying frontier topics for scholars to explore in more detail. (3) We conduct an interdisciplinary review and overcome the previous limitation of only focusing on a specific disciplinary perspective when conducting comprehensive analyses of social dilemma research issues. (4) We summarize the collaboration interests of different institutions, regions, and authors to provide a reference for researchers to identify potential partners. For example, scholars currently studying group size and group interaction in social dilemmas from the perspective of social physics may consider collaborating with Perc M, Wang Z, and Wang L.

The findings from our study provide practical solutions to real-world social dilemmas. Our research indicates that communication, procedural fairness, punishment, and reward can promote cooperation. Managers can leverage these findings to develop policies to foster cooperation among individuals facing social dilemmas. For instance, managers can enhance policy transparency and establish open communication channels to foster fairness and cooperation. By implementing feedback mechanisms, they can address concerns about fairness and equity. It is crucial to ensure that punishment is applied consistently and fairly to maintain credibility and effectiveness. Moreover, providing training and resources for effective communication, negotiation, and conflict resolution can further support these efforts. Managers can create an environment conducive to cooperative behavior by integrating these strategies into policy-making processes.

This study also has some research limitations: (1) we only analyzed English-language publications, overlooking literature in other languages. (2) This study overlooks other databases that include a wider range of journals, such as Scopus, Engineering Village, and PubMed. (3) We only analyzed academic papers, ignoring some nonacademic articles (e.g., government and corporate reports). (4) We have yet to consider additional articles published during the writing of this paper. (5) The scope of this research topic is broad, and it adopts a broad definition of concepts. However, the set topic keywords may only cover some articles in this research domain, resulting in sufficient accuracy in the research conclusions. (6) This article employed a network pruning algorithm to highlight important nodes identified by CiteSpace software. However, this approach may overlook some valuable articles, potentially leading to conclusions lacking universality.

In future research, we will include a variety of languages, the latest articles and works, and data sources to address these limitations, thereby enriching the analysis and improving the validity of the conclusions. We will also undertake a comparative analysis to examine the similarities and differences between the most recent research findings and the present conclusions, aiming to enhance the accumulation of knowledge in social dilemma research. Finally, to improve the completeness of the study, we will broaden the search scope and incorporate additional terms such as “public goods dilemmas” and “prisoner’s dilemma” in future research.

## Author contributions

**Conceptualization:** Yuqing Geng.

**Data curation:** Juan Gao, Jianyi Li.

**Formal analysis:** Juan Gao, Jianyi Li.

**Funding acquisition:** Juan Gao.

**Methodology:** Yuqing Geng.

**Project administration:** Yuqing Geng.

**Software:** Juan Gao.

**Supervision:** Yan Yan.

**Validation:** Juan Gao.

**Visualization:** Juan Gao.

**Writing – original draft:** Juan Gao.

**Writing – review & editing:** Xinying Jiang.
